# Phylogenetic survey of the subtilase family and a data-mining-based search for new subtilisins from *Bacillaceae*

**DOI:** 10.3389/fmicb.2022.1017978

**Published:** 2022-09-26

**Authors:** Fabian Falkenberg, Michael Bott, Johannes Bongaerts, Petra Siegert

**Affiliations:** ^1^Institute of Nano- and Biotechnologies, Aachen University of Applied Sciences, Jülich, Germany; ^2^Institute of Bio- and Geosciences, IBG-1: Biotechnology, Forschungszentrum Jülich, Jülich, Germany

**Keywords:** *Bacillaceae*, S8 protease family, subtilisin, data mining, subtilase, phylogenetic analysis

## Abstract

The subtilase family (S8), a member of the clan SB of serine proteases are ubiquitous in all kingdoms of life and fulfil different physiological functions. Subtilases are divided in several groups and especially subtilisins are of interest as they are used in various industrial sectors. Therefore, we searched for new subtilisin sequences of the family *Bacillaceae* using a data mining approach. The obtained 1,400 sequences were phylogenetically classified in the context of the subtilase family. This required an updated comprehensive overview of the different groups within this family. To fill this gap, we conducted a phylogenetic survey of the S8 family with characterised holotypes derived from the MEROPS database. The analysis revealed the presence of eight previously uncharacterised groups and 13 subgroups within the S8 family. The sequences that emerged from the data mining with the set filter parameters were mainly assigned to the subtilisin subgroups of true subtilisins, high-alkaline subtilisins, and phylogenetically intermediate subtilisins and represent an excellent source for new subtilisin candidates.

## Introduction

The subtilase family or subtilisin-like proteases defined by the MEROPS database as S8 family is the third largest family of serine proteases, both in terms of number of sequences and characterised peptidases, which are represented in microorganisms (archaea, bacteria, fungi, yeast) as well as in higher eukaryotes (Rawlings et al., 2014). The MEROPS database is a comprehensive source of information for proteases. It uses a hierarchical, structure-based classification, and based on statistically significant amino acid sequence similarities, proteases are grouped into families and clans (Rawlings et al., 2014). Here, the S8 family belongs to the clan SB, which is one of 13 clans of serine proteases and, in addition to the S8 family, also contains the S53 family (sedolisin family) (Page and Di Cera, 2008). The two families differ in their catalytic mechanism in that within S8 they form a catalytic triad with their active residues in the order Asp, His, Ser, referred to as the “classical” D-H-S family (Siezen et al., 2007). While protein folding of members of the family S53 is very similar to that of subtilisins, the catalytic triad has been altered to Glu, Asp, and Ser, which is referred to as the ED-S family (Siezen et al., 2007). Furthermore, the S8 family is subdivided into the two subfamilies S8A (subtilisin as a type example) and S8B (kexin as a type example) (Rawlings, 2020). In addition, subtilases were classified by Siezen and Leunissen (1997) into six groups based on sequence alignments of the catalytic domain, namely subtilisins, thermitases, proteinase K, lantibiotic peptidases, pyrolysins and kexins, while mentioning that further subdivision may become useful with more available sequences. The kexins form the subfamily S8B, while the other five groups belong to the subfamily S8A. In the MEROPS database, well-characterised specimens are selected and designated as “holotypes” at the subfamily level (Rawlings et al., 2014). There are currently 186 holotypes listed for S8A and 21 for S8B (Rawlings and Bateman, 2021). Uncharacterised homologs of a holotype were assigned to the same MEROPS identifier (Rawlings and Bateman, 2021). Here, only the catalytically active part of the protease (peptidase unit) is considered, and a new holotype is created when a protein is characterised that either has a different specificity than another protein in the subfamily or the same specificity but a different cellular location, has a different architecture, or the sequence in a phylogenetic tree does not cluster with that of an existing holotype with similar specificity (Rawlings and Bateman, 2021).

The known members of the S8 family are endopeptidases, with the exception of TPPII (tripeptidyl peptidase II), which releases tripeptides from the N-terminus of peptides (Siezen and Leunissen, 1997; Renn et al., 1998). In most bacteria, archaea, and lower eukaryotes they are mostly unspecific proteases and are involved in nutrition (Siezen and Leunissen, 1997). They fulfil other functions as well, as they are involved in developmental processes and immune responses in plants (Schaller, 2013), play a role in the metabolism of neuropeptides in *Drosophila melanogaster* (Renn et al., 1998), or are involved in pathogenesis (Garcia-Sanchez et al., 2004). Several subtilases contain a C-terminal extension, relative to the subtilisins, with additional properties such as sequence repeats, Cys-rich domains as cell surface anchors, or transmembrane segments (Siezen and Leunissen, 1997). Except for the subtilase ASP (*Aeromonas sobria* protease), an N-terminal propeptide acts as an intramolecular chaperone during maturation, supporting the folding of the catalytic domain (Zhu et al., 1989; Eder et al., 1993; Kobayashi et al., 2015). Members of the subtilase family find a wide range of applications in industry, such as lactocepins playing an important economic role in the industrial production of cheese and fermented milk (Broadbent and Steele, 2013). Of particular interest within this study are subtilisins, which find applications in several industrial sectors such as in detergents, leather processing, food, wastewater treatment, cosmetics, and pharmaceuticals (Kalisz, 1988; Solanki et al., 2021; Azrin et al., 2022). They are typically isolated from various species of the genus *Bacillus* such as *B. subtilis*, *B. licheniformis*, *Shouchella clausii* (formerly *Bacillus clausii*), etc. (Kalisz, 1988; Christiansen et al., 2003; Maurer, 2004; Joshi et al., 2021). They consist of about 270 amino acids and are secreted *via* the Sec-secretion pathway in a precursor form containing a signal peptide of about 28 amino acids and a propeptide of about 75 amino acids (Markland and Smith, 1971; Power et al., 1986; Siezen et al., 1991; Tjalsma et al., 2000).

Due to the increasing number of genome sequencing projects, the amount of data on uncharacterised proteins is growing exponentially (Rawlings, 2013). This rapidly increasing online database provides an excellent resource for broadening the sequence space of subtilisins. Our research on uncharacterised subtilisin sequences began with the analysis of the MEROPS S8 dataset in a phylogenetic tree containing only characterised proteases. The analysis quickly revealed the presence of previously uncharacterised groups and subgroups within the S8 family and motivated us to update the phylogeny of this family, which was necessary to place the uncharacterised sequences in this context. Sequences from a data mining approach for new subtilisin proteases from the *Bacillaceae* family were then evaluated and placed in the context of the S8 subfamilies and groups.

## Materials and methods

### Sequence-based phylogenetic analysis

The amino acid sequences of the mature part, referred to as the “peptidase unit” in the MEROPS database, comprising the structural domain of the protein directly responsible for peptidase activity and substrate binding, including the larger insertions compared to other subtilases (Rawlings et al., 2018). Other structural domains, if present, were excluded, such as the signal peptide, the propeptide and C-terminal domains. The sequences were aligned using MAFFT v 7.490 with L-INS-I parameter^1^ (Katoh and Standley, 2013; Katoh et al., 2019). The alignment was trimmed using trimAi v1.2 with the “gappyout” parameter^2^ (Sánchez et al., 2011). The phylogeny was made using iqtree v1.6.12^3^ (Trifinopoulos et al., 2016) with automated ModelFinder (Kalyaanamoorthy et al., 2017) and ultrafast bootstrap (Hoang et al., 2018) options. Phylogenetic trees were displayed and annotated with the iTOL software^4^ (Letunic and Bork, 2021).

### Data mining

Holotype protein sequences from the S8 family for the analysis were obtained from the MEROPS database^5^ (Rawlings et al., 2014). In addition, a selection of sequences of characterised subtilases was chosen from the Protein Data Bank (PDB). Only the mature part of the proteases was used as described above.

To search for uncharacterised subtilisin sequences from *Bacillaceae*, new amino acid sequences for the analysis were obtained from the NCBI Identical protein groups database^6^ by searching for “S8 peptidase *Bacillaceae*.” To selectively search for subtilisins, a filter was set for peptide sequences with a length of 350–410 amino acids. The resulting dataset was clustered with a identity threshold of 85% by using CD-HIT^7^ (Huang et al., 2010). Intracellular proteases were excluded by analysing the sequences with the SignalP 6.0 prediction tool^8^ and including only protein with a predicted Sec signal peptide (Teufel et al., 2022). The propeptide was removed manually after alignment with Clustal Omega^9^ (Sievers et al., 2011), using the JalView alignment annotation software^10^ (Waterhouse et al., 2009). MSA was drawn with ESpript 3.0 using %strict option (percentage of strictly conserved residues per column) for the colouring scheme^11^ (Robert and Gouet, 2014).

### Bioinformatic analysis

The isoelectric point of a protein was calculated with the sequence manipulation suite v2^12^ using pK_a_ values from DTAselect (Stothard, 2000).

## Results and discussion

### Phylogenetic tree analysis of the MEROPS S8 holotype dataset

**FIGURE 1:**
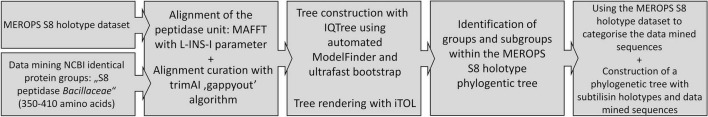
Workflow of used data and methods.

The first part of this study aimed at gaining a comprehensive overview of the S8A family in order to be able to categorise the sequences obtained from the data mining approaches in the second part. Therefore, a phylogenetic tree was first constructed containing only the MEROPS holotype dataset of the S8 peptidase unit and a selection of sequences of biochemically characterised proteases from the PDB with a total of 168 sequences. The additional PDB sequences were added to support some of the subfamilies described in literature. The amino acid sequences of the mature part, referred to as the “peptidase unit” in the MEROPS database, were used as described in methods. Other structural domains, if present, were excluded, such as the signal peptide, the propeptide and C-terminal domains. In uncurated datasets, it becomes difficult to identify N- and C-terminal extensions in a wide range of different sequences. During the alignment curation, trimAI reduced the alignment length to 248 positions as opposed to 2,330 positions without curation. Curation with trimAI, which uses a less stringent algorithm, was preferred to more stringent filtering methods, as these often result in the deletion of positions in the alignment that contain a gap (Tan et al., 2015). However, gaps can contain significant phylogenetic information (Dessimoz and Gil, 2010), not to mention that a sequence dataset may contain an incomplete or incorrect sequence, which can result in a large loss of information if left undetected. According to Tan et al. a less stringent filtering algorithm has little impact on tree accuracy and is a trade-off in terms of computation time saved for phylogenetic tree computation (Tan et al., 2015). An overview of the workflow of the analysis and the methods used is shown in Figure 1.

**FIGURE 2:**
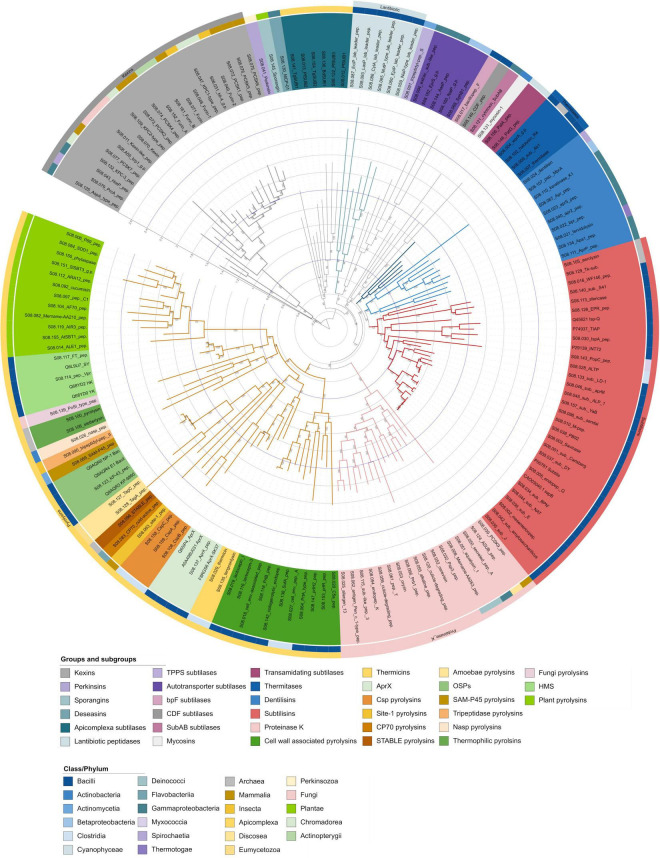
MEROPS S8 holotype phylogenetic tree. Phylogenetic relationship of S8 groups and subgroups using the mature protease sequences. The tree was constructed with IQ-TREE by employing the maximum likelihood method with ultrafast bootstrap support (model: LG + I + G4, predicted by Modelfinder, 1,000 replicates). The coloured range within the labels represents the subgroups as shown within the legend. The circles around the phylogenetic tree represent the class or phylum from which the holotype originates, marked by different colours. The kexin proteases S8B were selected as the outgroup. The outer ring represents the group classification after Siezen and Leunissen (1997). For each clade, the numbers above the branches indicate the bootstrap values based on 1,000 repetitions. The tree can be accessed under the following link: https://itol.embl.de/shared/2H14VxXLj30E2.

The curated alignment was used to create a maximum likelihood tree, the standard option for constructing phylogenetic trees, which, together with the Bayes method, is widely recognised as the most accurate approach in molecular phylogenetics (Kuhner and Felsenstein, 1994; Dereeper et al., 2008). In addition, it is important to statistically evaluate the reliability of the tree, which is usually done using a bootstrap-based bias correction method that calculates the branch support of the tree by repeating the tree construction (Hoang et al., 2018). However, the different parameters for the alignment, the curation methods, and the different tree generation methods result in different phylogenetic trees, making a detailed comparison of a generated tree with literature data difficult. Since the constructed tree is not rooted, an outgroup must be selected for restructuring the tree, which contains a set of sequences that are outside the ingroup but closely related to it (Sanderson and Shaffer, 2002). Figure 2 shows the phylogenetic tree of S8A subfamily, which is closely related to the S8B subfamily, which was selected as the outgroup (Siezen and Leunissen, 1997). In this tree, we identified the groups proteinase K, pyrolysins, thermitases, subtilisins, lantibiotic peptidases and kexins as described by Siezen and Leunissen (1997), and several subgroups within these groups. However, our analysis revealed that the S8A proteases form more groups and subgroups than previously described. To account for the diversity resulting from their different positions in the phylogenetic tree, their biochemical properties, biological functions, their structural similarity, and the taxa- and species-specific clusters formed, we propose a revision of the subtilase groups and smaller, better defined subgroups. The naming is based on the criteria mentioned if a connection is recognisable, otherwise they are named according to the protease first described in this group. These groups and subgroups are discussed below in order to place the subtilisins in the context of the subtilases and Table 1 provides an overview of them. Additionally, each group is shown as a pruned tree (Supplementary Figures 1–17).

**TABLE 1:** Group and subgroups of the subtilase family.

Group	Subgroup	References
Pyrolysins	Plant pyrolysins	Siezen and Leunissen, 1997
	High-mass subtilases (HMS)	Okuda et al., 2004
	Fungi pyrolysins	Faraco et al., 2005
	Thermophilic pyrolisins	Siezen and Leunissen, 1997
	Nasp pyrolysins	+
	Tripeptidase pyrolysins	Siezen and Leunissen, 1997
	SAM-P45 pyrolysins	+
	Oxidatively stable proteases (OSP)	Saeki et al., 2000
	Amoebae pyrolysins	+
	STABLE pyrolysins	+
	CP70 pyrolysins	+
	Site-1 pyrolysins	+
	Csp pyrolysins	+
	AprX	Phrommao et al., 2011
	Thermicins	+/ Jang et al., 2002
	Cell-wall associated pyrolysins	+
Proteinase K	Gram -	Siezen and Leunissen, 1997
	Fungal	Siezen and Leunissen, 1997
	Yeast	Siezen and Leunissen, 1997
Subtilisins	EPR subtilisins	+
	PopC subtilisins	+
	Extremophilic subtilisins	+
	Archaea subtilisins	+
	True subtilisins	Siezen and Leunissen, 1997
	High-alkaline subtilisins	Siezen and Leunissen, 1997
	Phylogenetically intermediate subtilisins (PIS)	Saeki et al., 2003
Dentilisins		+
Thermitases	Extremophilic thermitases	Siezen and Leunissen, 1997
Transamidating subtilases		Agarwal et al., 2012
Mycosins		Dave et al., 2002
SubAB subtilases		+
CDF subtilases		+
bpF subtilases		+
Autotransporter subtilases		Henderson and Nataro, 2001
TPPS subtilases		+
Lantibiotic peptidases		Siezen and Leunissen, 1997
Apicomplexa subtilases		+
Deseasins		Chen et al., 2007
Sporangins		+
Perkinsins		+
Kexins (S8B)	PC1	Siezen and Leunissen, 1997
	PC2	Siezen and Leunissen, 1997
	Furins	Siezen and Leunissen, 1997
	Yeast Kexins	Siezen and Leunissen, 1997

+Indicates that the group or subgroup was defined within this work.

### Pyrolysin group

The pyrolysin group clustered within the phylogenetic tree in 90% of the replicates. Pyrolysins are a heterogenous group of enzymes of diverse origin and low sequence conservation (Siezen and Leunissen, 1997). The average sequence identity of the pyrolysin group present in the phylogenetic tree is 38% (Figure 2). Within this branch, lactocepin 1 (S08.116) and lactocepin 3 (S08.019) are described by Siezen and Leunissen as pyrolysins in the gram-positive subgroup (Siezen and Leunissen, 1997; Broadbent and Steele, 2013). However, in the MEROPS phylogenetic tree, together with other sequences of gram-positive bacteria, they form a subgroup of their own, named here cell wall-associated pyrolysins with a bootstrap support of 100% and 51% sequence identity (Supplementary Figure 1). Lactocepins are cell envelope-associated endopeptidases of *Lactococci*, which play an important economic role in the industrial production of cheese and fermented milk due to their use as starter bacteria. Rapid growth is ensured by lactocepin, which provides amino acids from milk proteins and ensures autolysis, which is important for cheese ripening (Broadbent and Steele, 2013). They cluster together with other cell wall-associated proteases: S08.020 (Kagawa and Cooney); S08.153 (Pastar et al., 2003); S08.147 (Genay et al., 2009); S08.064 (Bethe et al., 2001); S08.027 (Lawrenson and Sriskandan); S08.138 (Karlsson et al., 2007); S08.118 (Gilbert et al., 1996). An exception is the thermophilic collagenolytic protease from *Geobacillus collagenovorans* MO-1 (S08.142), which contains a collagen-binding segment and is secreted into the culture supernatant without being displayed on the cell surface (Okamoto et al., 2001; Itoi et al., 2006).

Thermicin (S08.029), tengconlysin (S08.135), and the AprX proteases (S08.137) cluster together in the tree in 99% of the replicates (Supplementary Figure 2). Thermicin from the extremely thermophilic bacterium *Thermoanaerobacter yonseiensis* KB-1 (Jang et al., 2002) and tengconlysin from *Thermoanaerobacter tengcongensis* (Koma et al., 2007) show both high-temperature optima above 90°C. As mentioned by Jang et al. (2002) thermicin is a novel enzyme that differs from other thermostable proteases and here forms the new subgroup of thermicins. The Bacilli-derived AprX subtilases form another subgroup as previously described, because they lack a signal peptide and exhibit a mesophilic temperature optimum (Valbuzzi et al., 1999; Phrommao et al., 2011). The intracellular subtilase AprX-SK37 from *Virgibacillus* sp. SK37 is a halotolerant, oxidation-stable, and moderately thermophilic alkaline serine protease with properties that could be attractive for various biotechnological applications (Phrommao et al., 2011).

The three proteases CspB (S08.108), CspA (S08.159), and CspC (S08.158) (Clostridial serine proteases) from *Clostridium perfringens* form a new subgroup (Csp pyrolysins) with 45% sequence identity, related to germination and synthesised in the mother cell compartment of spore-forming cells (Supplementary Figure 3; Masayama et al., 2006).

Site-1 peptidase (S08.063) from *Cricetulus griseus* is an important processing enzyme of the endoplasmic reticulum/Golgi lumen that acts on sterol regulatory element binding proteins (SREBPs) to regulate cholesterol and fatty acid biosynthesis in addition to other cellular functions (Seidah, 2013b). CP70 (S08.083) from *Flavobacterium balustinum* is a cold-active extracellular protease (Morita et al., 1998); STABLE (S08.096) is a hyperthermostable protease bound to the surface layer of the archaeon *Staphylothermus marinus* and is responsible for the generation of the peptides required in the energy metabolism of the cell (Mayr et al., 1996). Because of their different physiological functions and origins, they will most likely all form new individual subgroups as more homologs are added (Site-1 pyrolysins, CP70 pyrolysins, STABLE pyrolysins). TagA (S08.128) and TagC (S08.127) are produced by the amoeba *Dictyostelium discoideum*. While TagA is involved in the differentiation of cell types (Good et al., 2003), TagC is part of a transmembrane protein of the ABC family, which is expressed during the aggregation stage of development (Anjard and Loomis, 2005). TagA and TagC are forming the new subgroup of amoebae pyrolysins with a 100% bootstrap support and 42% sequence identity (Supplementary Figure 3).

KP-43 (S08.123) from *Bacillus* strain KSM-KP43 and the sequences within this clade form the subgroup of oxidatively stable serine proteases (OSPs) as described by Saeki et al., with a sequence identity of 92% (Saeki et al., 2000, 2002). However, it should be mentioned that subtilisins not belonging to this subgroup were reported to have higher stability against H_2_O_2_ (Joshi and Satyanarayana, 2013; Falkenberg et al., 2022). The C-terminal half of KSM-KP43 downstream of the putative catalytic residue, Ser-255, is homologous to the internal segments of TagC (Saeki et al., 2002). While the MEROPS dataset suggests that OSPs are only of bacterial origin, Li et al. (2017) described sequences originating from some species of *Pezizomycotina* fungi.

SAM-P45 (S08.069), a membrane-anchored protease from *Streptomyces albogriseolus* which is considered to be an evolutionary link between primitive bacterial subtilisins and highly diversified eukaryotic proteases, forms its own new subgroup (SAM-P45 pyrolysins) (Suzuki et al., 1997). TPPII, isolated from *Drosophila melanogaster*, has an elongated C-terminus compared to other subtilases and is involved in the metabolism of neuropeptides, which are important signalling molecules in insects and belongs to the tripeptidase pyrolysins subgroup (Siezen and Leunissen, 1997; Renn et al., 1998). The extracellular serine protease Nasp (S08.026) from *Dermatophilus congolensis* is involved in pathogenesis and forms the new subgroup Nasp pyrolysins (Garcia-Sanchez et al., 2004). The two thermophilic proteases from archaea *Thermococcus stetteri* (stetterlysin; S08.106) (Klingeberg et al., 1995) and *Pyrococcus furiosus* (pyrolysin; S08.100), the eponym of the whole group (Blumentals et al., 1990) are both resistant against SDS (1% w/v) and have a high-temperature optimum (85°C, 115°C) forming the thermophilic pyrolysins subgroup (Supplementary Figure 4; Siezen and Leunissen, 1997).

PoSI (*P. ostreatus* extracellular protease) (S08.139), a protease from the fungus *Pleurotus ostreatus*, is involved in the activation of other secreted proteases and the post-translational regulation of laccase (Faraco et al., 2005). It shows a high sequence identity with the minor extracellular serine protease from *Bacillus subtilis*, Vpr (S08.114) (31%), and together with other proteases from Ascomycetes and Basidiomycetes forms a separate pyrolysin subgroup (fungi pyrolysins) (Supplementary Figure 5; Faraco et al., 2005). A further subdivision of fungal subtilases has been made by others and is beyond the scope of this study (Hu and Leger, 2004; Muszewska et al., 2011; Li et al., 2017).

According to Okuda et al. Vpr (S08.114) belongs to the subgroup of high-molecular-mass subtilisins, which can be divided into at least two classes (Supplementary Figure 5; Okuda et al., 2004). One class is less alkaline, its stability depends on Ca^2+^ ions and it is resistant to proteolysis. The other class is strongly alkaline, its stability also depends on Ca^2+^ ions and is sensitive to proteolysis (Okuda et al., 2004). The subgroup name is slightly misleading, as they do not cluster together with the subtilisins. Therefore, they were named here high-molecular-mass subtilases (HMS). Here, the average sequence identity is 82%.

Plant subtilases are a widely distributed subgroup involved in plant developmental processes and immune responses (Schaller, 2013). The average sequence identity between the investigated sequences is 56% (Figure 2 and Supplementary Figure 6). The first subtilase cloned from plants was the extracellular alkaline protease cucumisin (S08.092) from melon fruit (Kaneda and Tominaga, 1975). The plant subtilases have been divided into seven classes (Xu et al., 2019). A detailed discussion of each of these classes is beyond the scope of this study. Good overviews of the classes and the plant subtilases were provided by Schaller (2013), Taylor and Qiu (2017), and Xu et al. (2019).

### Proteinase K group

The alkaline proteinase secreted into the culture medium by the mould *Tritirachium album* Limber is commonly known as proteinase K (S08.054) and is the type example for this group (Ebeling et al., 1974). It can be used to synthesise peptides (Ageitos et al., 2013), and besides peptide bonds, it can also hydrolyse esters (Borhan et al., 1996). In contrast to subtilisins, which contain no cysteine residues, proteinase K contains five Cys residues, four which form two disulfide bridges (Betzel et al., 1990). Because of its remaining activity at higher temperatures (> 60°C) in the presence of urea, 0.5% (w/v) SDS, or 1% (w/v) Triton X100, proteinase K is used for the degradation of proteins and in the preparation of nucleic acids (Sweeney and Walker, 1993; Goldenberger et al., 1995). Most of the proteinase K holotypes found in the phylogenetic tree derive from fungi (Figure 1). Worlflow of used data and methods.

There, the fungal proteinase K subgroup is separated from the other proteinase K members and may play an important role in the evolution of pathogenicity, as several entomopathogenic and nematophagous fungi have been characterised as having the ability to destroy the structural integrity of insect or nematode cuticle during invasion and colonisation. Therefore, they are also referred to as cuticle-degrading proteases (S08.120, S08.056) (Leger et al., 1987; Tunlid and Jansson, 1991; Li et al., 2010). For many saprophytes, the subtilases as broad-spectrum proteases play a role in nutrition acquisition, such as digesting proteins to release peptides and amino acids (Gunkel and Gassen, 1989; Hu and Leger, 2004). Further phylogenetic analysis by Li et al. of 138 fungal proteinase K genes revealed a subdivision into five distinct classes (Li et al., 2017). The fungal proteinase K-like proteases are separated from the bacterial ones including aqualysin (S08.051) from the thermophilic bacterium *Thermus aquaticus* (Sakaguchi), the *Amoebozoa* protease ASUB (S08.124) from *Acanthamoeba healyi* (Kong et al., 2000), and the proprotein convertase PCSK9 (S08.039) from *Mus musculus* (Seidah, 2013a; Supplementary Figure 7). The average sequence identity within the proteinase K family in the phylogenetic tree is 48%.

### Thermitase/subtilisin group

Thermitase, the type enzyme for this group, is an extracellular, thermostable protease of the thermophilic microorganism *Thermoactinomyces vulgaris* (Frömmel et al., 1978). For an in-depth review of this protease see Betzel (2004). The other three enzymes of the thermitase-type WprA (S08.004) (Margot and Karamata, 1996), halolysin (S08.102) (Kamekura et al., 1996), and Subtilisin AK1 (S08.009) (Toogood et al., 2000) were also identified by Siezen and Leunissen (1997) and forming the subgroup of extremophilic thermitases. While halolysin derives from the halophilic archaeon *Haloferax mediterranei* (Kamekura et al., 1996), all other members come from Bacilli (see Supplementary Figure 8 and Figure 2). Figure 2 (light blue) shows a more distinctive group with two clades. Here, Siezen and Leunissen identified bpr (S08.022) of *Dichelobacter nodosus* as a thermitase and AprP of *Pseudomonas sp*. KFCC 10818 as a subtilisin (Lilley et al., 1992; Jang et al., 1996; Siezen and Leunissen, 1997). However, the bootstrap value for these two clades is only 18%, which is why they are treated as an intermediate subgroup between thermitases and subtilisins. This intermediate new subgroup is named here as dentilisins, since this protease was already described in 1990 (Que and Kuramitsu, 1990). In general, thermitases are co-located in the clade with subtilisins, highlighting their similarity.

The subtilisin Carlsberg (S08.001) is the type example of the subtilisins and the entire S8 family and belongs to the subgroup of true subtilisins, along with the high-alkaline subtilisins, the intracellular subtilisins, and the phylogenetically intermediate subtilisins (PIS) (Smith et al., 1966; Siezen and Leunissen, 1997; Saeki et al., 2003). Extracellular subtilisins play an important role in nutrition, whereas intracellular subtilisins (Isp), such as IspA (S08.030), play a role in protein turnover and processing during sporulation or are involved in the heat shock response (Reysset and Millet, 1972; Koide et al., 1986). As shown in Figure 2 all holotypes are derived from microorganisms, mainly from Bacilli, while aerolysin (S08.105) (Völkl et al., 1994), Tk-subtilisin (*Thermococcus kodakaraensis* subtilisin) (S08.129) (Kannan et al., 2001), PopC, (S08.143) (Rolbetzki et al., 2008), and ALTP (*Alkaliphilus transvaalensis* protease) (S08.028) (Kobayashi et al., 2007) derive from Archaea, Myxococci, and Clostridia, respectively. However, *Bacillus* as the most prominent source of subtilisins spawned alkaline proteases such as subtilisin Carlsberg, BPN’, and Savinase, which have their major application as detergent enzymes with excellent properties, including high stability toward extreme temperatures, pH, organic solvents, detergents, and oxidising compounds (Kalisz, 1988; Contesini et al., 2018). Besides the application in detergents, subtilisins are applied for example in leather processing, food, wastewater treatment, and cosmetics (Kalisz, 1988; Solanki et al., 2021). These subtilisin holotype sequences will be used for the classification of the new sequences provided by a data mining approach.

### Various diverse groups

Several holotypes form a clade together within the phylogenetic tree (Figure 2 and Supplementary Figure 9). Due to their different origins, their different biological functions, and their low bootstrap value (40%), they most likely form individual groups. Bacillopeptidase F (bpF) (S08.017) from *B. subtilis* is a cell envelope protein and contributes to nutrition in the soil environment (bpF subtilases) (Hageman). While Siezen and Leunissen grouped bpF within the pyrolysins, it is separated in this study, which could be due to the fact that Siezen and Leunissen only analysed the amino acids around the catalytically active ones (Siezen and Leunissen, 1997). CDF (S08.149) from Thermoactinomyces sp. CDF is a protease located on the surface of the spore coat (CDF subtilases) (Cheng et al., 2009). Cytotoxin SubAB (S08.121) is a toxin from Escherichia coli with two subunits, where subunit B binds to the surface receptor of target cells and subunit A, the enzymatically active moiety, is responsible for cytotoxicity and has a very narrow substrate specificity (SubAB subtilases) (Yahiro et al.).

Mycosin-1 (S08.131) from *Mycobacterium tuberculosis* is an extracellular protein that is membrane- and cell wall-associated and is expressed after infection of macrophages, forming the group of mycosins (Dave et al., 2002). PatA (S08.156) and PatG (S08.146) from *Prochloron didemni* are involved in the maturation of cyanobactins in *Cyanobacteria* and form the group of transamidating subtilases (Lee et al., 2009; Agarwal et al., 2012).

A separated clade can be also observed for the new group of tripeptidyl peptidase subtilases (TPPS) and the group of autotransporter subtilases (AT) (Henderson and Nataro, 2001; Supplementary Figure 10). Like tripeptidyl peptidase II (TPPII, S08.090) from the pyrolysin group, tripeptidyl peptidase S (TPPS) (S08.091) from Streptomyces lividans is an exopeptidase that cleaves tripeptide units from oligopeptides or polypeptides and probably forms its own group (Butler, 2013). The extracellular *Serratia* serine protease (SSP) (S08.094) from *Serratia marcescens* (Yanagida et al., 1986) was grouped by Siezen and Leunissen (1997) to gram-negative pyrolysins. However, SSP together with EprS (S08.162) (Kida et al., 2013), AasP (autotransported serine protease A) (S08.1449) (Ali et al., 2008), NalP (Neisserial autotransporter lipoprotein) (S08.160) (Turner et al., 2002), and SphB1 (S08.068) (Coutte et al., 2001), are forming the group of autotransporter subtilases in gram-negative bacteria with a low sequence identity of 35%.

An additional clade with 94% bootstrap support is formed by perkinsin (S08.041) from the protist *Perkinsus marinus*, which is an enzyme of unknown function but may be involved in cell invasion of the eastern oyster *Crassostrea virginica* (Brown and Reece, 2003; Supplementary Figure 11). Sporangin (S08.145) from the alga *Chlamydomonas reinhardtii* is localised to the flagella of daughter cells within the sporangial cell wall and is released into the culture medium where it is involved in the digestion of the sporangial cell wall (Kubo et al., 2009). Perkinsin and sporangin are forming two new groups (perkinsins, sporangins). MCP-01 (S08.130), the extracellular cold-adapted protease from the deep-sea bacterium *Pseudoalteromonas* sp. SM9913 forms the group of deseasins secreted mainly by bacteria in deep-sea or lake sediments (Chen et al., 2007; Zhao et al., 2008). It is a multidomain protein with a collagen-binding domain at its C-terminus that exhibits collagenolytic activity and therefore plays an important role in the degradation of particulate organic nitrogen from deep-sea sediments (Zhao et al., 2008). The proteases produced by the parasites in the phylum Apicomplexa are forming a new group (Apicomplexa subtilases) with TgSub1 (Toxoplasma gondii) (S08.141) (Miller et al., 2001), PfSUB2 (Plasmodium falciparum) (S08.013) (Hackett et al., 1999), TgSUB2 (S08.154) (Miller et al., 2003), BdSUB1 (Babesia divergens) (S08.136) (Montero et al., 2006), PfSUB3 (S08.122) (Withers-Martinez et al., 2004), and PfSUB1 (S08.012) (Withers-Martinez et al., 2004), with a sequence identity of 34%. These proteases are involved in the host-cell invasion (Silmon de Monerri et al.).

### Lantibiotic peptidase group

The lantibiotic peptidase group within S8A subfamily comprises highly specialised enzymes for cleavage of leader peptides from precursors of the antimicrobial peptides (lantibiotics) (Sahl et al., 1995; Supplementary Figure 12). ElkP (S08.095) and PepP (S08.85) from *Staphylococcus epidermidis* are not included in the dataset because only sequence fragments were available. Lantibiotic peptidases are found intracellularly, extracellularly and membrane-anchored (Bierbaum et al.). The sequences included in the phylogenetic tree show 31% sequence identity.

### Kexin subfamily (S8B)

Kexin the type example for the subfamily S8B and was first identified in *Saccharomyces cerevisiae*. It can process the yeast precursors of alpha-mating factor and killer toxin and plays a significant role in post-translational modification in eukaryotes (Rogers et al., 1979). For a review see Fuller (2013). AspA (S08.125) is one of the two subtilases which is lacking a propeptide and stands apart within the S8B family (Figure 2; Mellergaard, 1983; Kobayashi et al., 2015). Siezen and Leunissen (1997) also mentioned that AspA is a more distant member. As mentioned above, the S8B subfamily was used as an outgroup for the phylogenetic tree as it is closely related to the S8A subfamily (Siezen and Leunissen, 1997). Within the phylogenetic tree, it forms a clearly defined clade comprising all 21 holotype sequences of the MEROPS S8B subfamily, with an average sequence identity of 53% (Supplementary Figure 13). The clustering with a bootstrap support of 89% supports that kexins are a distinct subfamily (S8B) within the subtilase subset (Siezen and Leunissen, 1997). Kexins are also divided into at least four subgroups: PC1, PC2, furins and yeast kexins, but the subdivision will not be discussed further here as the focus is on the S8A subfamily (Siezen and Leunissen, 1997).

### Data mining and phylogenetic tree analysis of subtilisins from *Bacillaceae*

Due to the increasing number of genome sequencing projects, the amount of data on uncharacterised proteins is growing exponentially (Rawlings, 2013). Many genomes encode multiple secreted proteases and many proteases can be found in different species (Takimura et al., 2007). The great potential of the data mining approach becomes clear when looking at the huge number of 247.897 hits (January 31, 2022) that were found when searching for S8 peptidases within the NCBI identical protein groups database. Within this second part of our study, we focused on subtilisins derived from Bacillaceae because they are right now the most relevant industrial proteases (Maurer, 2004; Azrin et al., 2022).

**FIGURE 3:**
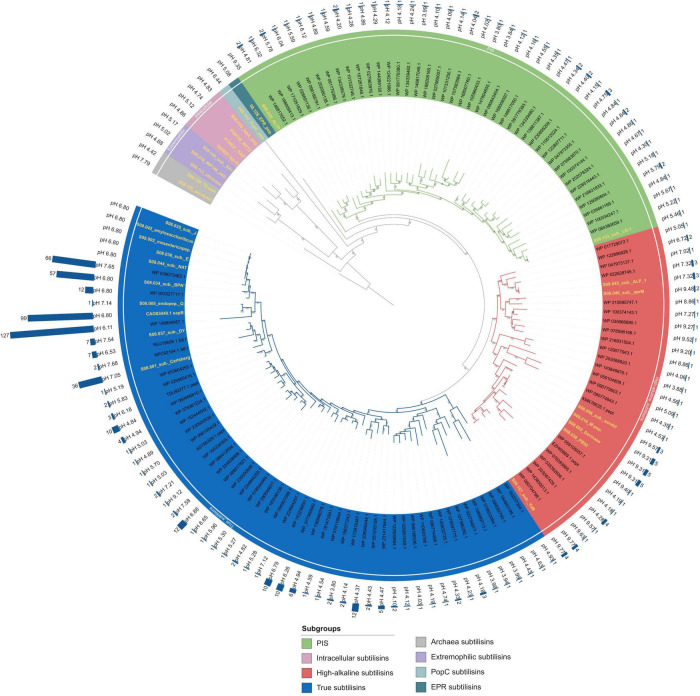
Phylogenetic tree of 120 novel subtilisins from *Bacillaceae* identified by a database search. The tree was constructed with IQ-TREE using the maximum likelihood method with ultrafast bootstrap support (model: LG + I + G4 predicted by Modelfinder, 1,000 replicates). The coloured area inside and outside the labels represents the subgroups, as indicated in the legend. The theoretical isoelectric point is given for each sequence in the outer circle. Aerolysin was chosen as the outgroup. Archaea subtilisins, extremophilic subtilisins, EPR – and PopC subgroup were added as additional holotypes. All holotypes are highlighted with a yellow text colour. The bar graphs represent the cluster size of the data mining search. For each group, the numbers above the branches indicate the bootstrap values based on 1000 replications. The tree can be accessed at the following link: https://itol.embl.de/shared/2H14VxXLj30E2.

To search for new subtilisin sequences from *Bacillaceae*, the database search was performed as described above and in Figure 1. The search yielded 1,424 sequences with the set values. With the length specification of 350–410 amino acids, sequences typical of AprX, lantibiotic peptidases, kexins, OSP, and HMS are excluded, while typical thermitases, intracellular subtilisins, proteinase K, and high/true/PIS subtilisins can still be found. The size exclusion was set to reduce the number of sequences (18.881 without size exclusion) and was chosen because typical subtilisin sequences derived from *Bacillaceae* are around 380 amino acids long, including the signal peptide and the propeptide (Markland and Smith, 1971; Power et al., 1986; Siezen et al., 1991; Tjalsma et al., 2000). Without the size exclusion, many additional new subtilases from *Bacillaceae* could probably be found. CD-HIT clustering with an identity threshold of 85% yielded 375 clusters. For each cluster, one representative was used for further analysis. The number of sequences within one cluster is displayed as a bar chart around the phylogenetic tree in Figure 3. Signal peptide analysis identified 135 sequences without a signal peptide, reducing the dataset to 240 sequences, as we are only interested in extracellular proteases for further analysis and potential biochemical characterisation. The remaining sequences were aligned and the propeptide was manually removed as described above. Sequences that could be directly visually classified as thermitases after alignment were discarded, leaving a sequence set of 120 sequences within the sequence space of subtilisins as shown in Figure 3 (Supplementary Table 1). Sequences from the first phylogenetic tree comprising all 168 MEROPS holotypes, which build the subfamilies of subtilisins, were used again and aligned with the 120 sequences from the data mining approach. The sequence alignment was refined with TrimAI, which reduced the alignment length to 260 positions in contrast to 448 positions without refinement. Here, the two archaea subtilisins were used as an outgroup to reroot the tree. Figure 3 shows that all sequences derived from *Bacillaceae* in the data mining set represent the three main subgroups within the subtilisins, the true subtilisins, the high-alkaline subtilisins, and the phylogenetically intermediate subtilisins.

In addition to the above mentioned three subgroups, the following proteases form additional subgroups (Supplementary Figure 14): Aerolysin (S08.105) and Tk-subtilisin (S08.129) from the hyperthermophilic archaea *Pyrobaculum aerophilum* (Völkl et al., 1994) and *Thermococcus kodakaraensis* (Kannan et al., 2001) are forming an own new subgroup (Archaea subtilisins) with a sequence identity of 47%. Subtilisin S41 (S08.140) a psychrophilic protease from antarctic *Bacillus* TA41 (Almog et al., 2009), WF146 (S08.016), a thermophilic protease from *Bacillus sp.* WF146 (Wu et al., 2004), and Sfericase (S08.113), a psychrophilic protease from *Lysinibacillus sphaericus*, are forming a new subgroup named here extremophilic subtilisins with a sequence identity of 70%. The fact that no similar sequences from the group of extremophilic subtilisins were found in the data mining search could be due to the fact that all three representatives are larger than 410 amino acids, which also applies to EPR (S08.126), an extracellular protease from *B. subtilis* involved in cell motility (Dixit et al., 2002). PopC (S08.143) is involved in the cell signalling cascade for forming *Myxococcus xanthus* cells into fruiting bodies and sporulation (Rolbetzki et al., 2008). EPR and PopC are forming two new individual subgroups (EPR subtilisins, PopC subtilisins). The intracellular subtilisins form their own known subgroup with a sequence identity of 72% (Siezen and Leunissen, 1997). Since all sequences without signal peptides were excluded from the data mining set, no sequences are clustered with the holotypes. In general, subtilisins are mainly found in Bacilli and none in fungi (Siezen et al., 2007; Muszewska et al., 2011). In the following, all amino acid positions refer to the BPN’ numbering.

The subgroup of **true subtilisins** includes subtilisin Carlsberg (S08.001) from *Bacillus licheniformis* (Linderstrøm-Lang and Ottesen, 1947; Güntelberg and Ottesen, 1952; Smith et al., 1966), which toghether with BPN’ (S08.032) from *Bacillus amyloliquefaciens* were the first two subtilisins to be studied in detail (Matsubara et al., 1965; Smith et al., 1966). Their group is supported by a 91% bootstrap value within the phylogenetic tree (Figure 3 and Supplementary Figure 17). Interestingly the data mining search revealed the most similar sequences within the subgroup around these holotypes, as indicated by the bar chart with cluster sizes up to 127 sequences. Several newly found sequences are phylogenetically more distinct from any known holotype, which suggests that these sequences could have other biochemical characteristics and may form new classes. The calculated isoelectric point of the sequences within the true subtilisins is on average rather acidic to neutral. The representatives of this subgroup characterised so far are more sensitive and less active under high-alkaline conditions compared to the high-alkaline subtilisins (Nakamura et al., 1973; Maeda et al., 2001).

The subgroup of **phylogenetically intermediate subtilisins** (PIS) was introduced by Saeki et al. with the biochemical characterisation of the subtilisin LD1 (S08.133) from the alkaliphilic *Bacillus* sp. KSM-LD1. Due to its properties and the phylogenetic position, LD1 forms a subgroup at an intermediate position between true subtilisins and high-alkaline subtilisins (Saeki et al., 2003). LD1 has a C-terminal extension of 29 amino acids, suggesting an association with the cell surface of *Bacillus* sp. KSM-LD1 (Saeki et al., 2003). Interestingly, the sequences WP_100334247.1 from *Bacillus alkalisoli* and WP_084380659.1 from *Sutcliffiella cohnii* have a C-terminal extension like LD1 (Spanka and Fritze, 1993; Liu et al., 2019). LD1 and other PIS have multiple amino acid insertions compared to BPN’, but this does not affect substrate specificity toward synthetic substrates (Saeki et al., 2003). The protease ALTP (S08.028) from the anaerobic and extremely alkaliphilic *Alkaliphilus transvaalensis* is the first high-alkaline protease reported from a strict anaerobe (Kobayashi et al., 2007). ALTP is 66% identical to LD1 and, according to Kobayashi et al., it is in an intermediate position between the true and the highly alkaline subtilisins (Kobayashi et al., 2007). This assignment is supported by the phylogenetic tree (Figure 2) constructed in our study. In the phylogenetic tree with the newly mined database sequences (Figure 3 and Supplementary Figure 15), ALTP is separated from the *Bacillaceae*-derived phylogenetically intermediate subtilisins, as it is derived from the bacterial class *Clostridia.* ALTP has solely an alkaline isoelectric point, while the other sequences within this subgroup all have an acidic pI (Figure 3).

The subgroup of **high-alkaline subtilisins** was discovered in the 1980s and originates from alkaliphilic *Bacilli* (Ito et al., 1998; Maurer, 2004). Since the first discovery of protease no. 221, an increasing number of high-alkaline subtilisins have been characterised (Nakamura et al., 1973). Alkaline subtilisins, such as Savinase, are much more stable in an alkaline environment than true subtilisins such as BPN’ or subtilisin Carlsberg and can be used to adapt to harsh industrial conditions, especially in modern detergents (Maurer, 2004). Within the phylogenetic tree they are forming a distinct subgroup with a branch support of 93% (Figure 3 and Supplementary Figure 16). ALP-1 (S08.045) from *Bacillus* sp. NKS-21 (Yamagata et al., 1995), WP_017729072.1 from *Halalkalibacterium ligniniphilum* (Zhu et al., 2014; Joshi et al., 2021), WP_122896828.1 from *Alteribacter keqinensis* (Liu et al., 2022), WP_047973137.1 from *Bacillus* sp. LL01 (Vilo et al., 2015), and WP_022628745.1 from *Alkalihalophilus marmarensis* (Denizci et al., 2010; Joshi et al., 2021) form a more separated clade, as they lack the four amino acid deletion around position 160, in contrast to the other high-alkaline proteases. This position corresponds to a loop near the P1 binding site (Wells et al., 1987; Betzel et al., 1992). They form another class of the ALP-1-type subtilisins, as mentioned by Yamagata et al. (2002). Additionally, the theoretical isoelectric point of these proteins is neutral to acidic in contrast to the majority of the other high-alkaline proteases. High-alkaline proteases adapt to higher alkaline conditions by an altered surface charge at higher pHs, as indicated by an increased pI value caused by a higher number of Arg and a decreased number of Lys residues (Masui et al., 1998). The substrate specificity of ALP-1 toward the B-chain of insulin differs from that of other alkaline subtilisins, but is similar to that of neutrophilic subtilisins, which may be related to the deletion of four amino acids around position 160 (Tsuchida et al., 1986; Yamagata et al., 1995). For ALP-1, an enzyme engineering study identified amino acids in the C-terminal region that increased stability 120-fold under alkaline conditions after replacement (Yamagata et al., 2002). Some of the high-alkaline proteases including Savinase have an extra proline at position 131, which provides extra active-site rigidity compared with other subtilisins (Betzel et al., 1996). Recently, we reportet about SPAO from *Alkalihalobacillus okhensis* Kh10-101*^T^*, which showed high stability against hydrogen peroxide and NaCl concentrations up to 5.0 M (Falkenberg et al., 2022). SPAO can be assigned here to the holotype subtilisin sendai (S08.098) (Figure 3).

The average sequence identity between the sequences within the three subgroups of high-alkaline, PIS, and true subtilisins was calculated to be 67, 72, and 66%, respectively (sequences from Figure 3). The identity between true and high-alkaline subtilisins is 58%, between true and PIS 57%, and between high-alkaline subtilisins and PIS 55%.

A detailed investigation of all insertions and deletions within the three subgroups PIS, high-alkaline, and true subtilisins showed that the four amino acid deletion in the clade of aprM (S08.046) (Takami et al., 1990; Masui et al., 1994) is between Ser^161^ and Thr^174^, while for the other high-alkaline proteases, except for the ALP-1 clade mentioned above, the deletion is between Gly^160^ and Thr^174^ (Supplementary Figure 18). Interestingly, all high-alkaline subtilisins have a deletion of one amino acid at positions 37 and 57 (Supplementary Figure 18). The loop of amino acids 50–59 is known to be one of the most variable parts of subtilisin structures (van der Laan et al., 1992). Therefore, deletions within this loop could be detected in several sequences within the three subfamilies. Several PIS sequences have a double insertion between positions 42 and 43 in common. All sequences within the PIS subgroup share the insertion between positions 159 and 160, while high-alkaline subtilisins have a deletion of four amino acids around this position. Position 160 is localised in a loop that, as mentioned above, takes part in the conformation of the P1 pocket and might be involved in the P1 preference, and the recognition of steric conformation (Yamagata et al., 1995). Additionally, shorter loops can increase the stability of an enzyme (Gavrilov et al., 2015). In general, all insertions or deletions are located at the surface of the protease, which could be due to the fact that the overall structure within the subtilisins is highly conserved (Goddette et al., 1992).

For a distinct further subdivision of true subtilisins, high-alkaline subtilisins and PIS into classes, supporting biochemical data might be necessary. However, based on the phylogenetic tree, the deletion and insertion analysis, and the isoelectric point, a further subdivision into classes is most likely.

## Conclusion

Phylogenetic studies of the S8 family within the MEROPS holotype dataset revealed a large number of different subtilases forming new groups and subgroups. In addition to the known groups of proteinase K, pyrolysins, kexins, subtilisins, thermitases and lantibiotic peptidases, the analysis revealed new groups or subgroups within the S8A subfamily depending on their position in the phylogenetic tree, their biochemical properties or their origin. This analysis was used in the second part of this study to categorise 120 newly identified predicted S8 protease sequences derived from *Bacillaceae*. They were found to represent the three main subgroups within the subtilisins, the true subtilisins, the high-alkaline subtilisins, and the phylogenetically intermediate subtilisins. However, without the specified filter parameters for data mining, more new subtilases outside the group of subtilisins from *Bacillaceae* could probably be found. In the absence of experimental characterisation for most of the found subtilisin sequences, a subdivision needs further experimental studies, because with bioinformatic analysis alone, a prediction of their biological and biochemical properties is possible only to a limited extent. For the newly found enzymes it is thus possible that they possess unique specificities and are of high interest for biotechnological applications.

## Data availability statement

The original contributions presented in this study are included in the article/Supplementary material, further inquiries can be directed to the corresponding author.

## Author contributions

FF collected and analyzed the data and wrote the original draft. JB, MB, and PS supervised the study and revised the manuscript. All authors contributed to the final manuscript.

## Footnotes

1
https://mafft.cbrc.jp/alignment/server/

2
http://phylemon.bioinfo.cipf.es/

3
http://iqtree.cibiv.univie.ac.at/

4
https://itol.embl.de/

5 https://www.ebi.ac.uk/merops; published before January 19, 2022.

6 https://www.ncbi.nlm.nih.gov/ipg; published before January 31, 2022.

7
http://weizhong-lab.ucsd.edu/cdhit-web-server

8
https://services.healthtech.dtu.dk/service.php?SignalP-6.0

9
https://www.ebi.ac.uk/Tools/msa/clustalo/

10
https://www.jalview.org/

11
https://espript.ibcp.fr/ESPript/ESPript/

12
https://www.bioinformatics.org/sms2
