# Supplementary material for: Phylogenetic survey of the subtilase family and a data-mining-based search for new subtilisins from Bacillaceae
Source: Front Microbiol. 2022 Sep 26;13:1017978. doi: 10.3389/fmicb.2022.1017978 (PMC9549277; doi:10.3389/fmicb.2022.1017978)
Supplement: Supplementary file 1 [file Data_Sheet_1.pdf]

```
                                     1         10        20        30        40        50
                                     .         .         .         .         .         .
WP_105960433.1                       NQEEPYGIGQIQSDEAY.ELGVDGAGMNVAVLDTGIDASHEDLEGNVVDGYSVFTDSENS
WP_107583584.1                       GQTVPYGIPQIQSIETQ.QEGFTGEGVSVAVLDTGIDGSHEDLVDNLAGGYSVFTDAENA
WP_168006597.1                       LQDIPYGIESVQGVQAQ.ELGFRGDGMDVAILDTGIDINHEDLAANVQGGFSVFSDSANS
WP_090843404.1                       LQDIPYGIEQVQSLDVQ.GLGFRGQGMSVAVLDTGIDINHQDLYDNIQGGYSVFTDSANS
WP_147804655.1                       AQDVPYGIDQVQSTDAQ.AQGIEGDGMSVAVLDTGIDASHEDLADNVVDGYSVFTDSENS
WP_168007760.1                       SQTVPYGIDQVQATDAY.AQGISGDGVSVAVLDTGIDADHEDLADNVVGGYSVFSDADNS
WP_146817052.1                       YQDVPYGIQSVQATDVH.PFGYYGQGVDVAILDTGIDASHEDL..NVSGGHSVFSNWPES
WP_026691136.1                       SQTTPYGVPQVQATDVH.QYGYFGQGVRVAVLDTGIDANHSDL..NVYGGHSVFSNWPES
WP_171051829.1                       SQTTPYGVSQVQAPDVH.QYGYFGQGVRVAILDTGIDSSHQDL..NVHGGHSVFSNWPES
WP_168009413.1                       MQTVPYGISQVQAPDVH.RFGYFGQGVRVAILDTGIDANHQDL..NVYGGHSVFSSWPDS
WP_124221886.1                       SQDTPWGIPHVQGTEAQ.DNGHTGEGVKVAVLDSGIDASHEDL..NVADGYSSVDDS...
WP_134339482.1                       AQQTPWGIPHVEGTTAQ.NNGHTGDGVKVAILDTGIDASHEDL..VVAGGYSVFGDS...
WP_091776380.1                       AQETPWGVPHVQATEAQ.SNGSTGDGVKVAILDSGVDANHEDL..EVAGGHSVFGDS...
WP_100334247.1                       AQTVPWGVTHVQATEAH.ASGHTGSGIKVAVLDTGIDRNHLDL..NVRGGHSVFTDSANN
S08.133_sub._LD-1                    AQTVPWGVPHVQGTDAH.AAGHTGSGVKVAILDTGIDRNHEDL..NVRGGHSVFTDSANR
WP_084380659.1                       AQTVPWGVPHVQGTDAH.AAGHTGSGVKVAILDTGIDRNHEDL..NVRGGHSVFTDSANS
WP_134339480.1                       AQTTPWGVPHVQGTDAQ.NAGYTGNGVKVAILDTGIDATHEDL..TVSGGHSVFDDAENS
WP_091776383.1                       AQTTPWGISHVQGTEAQ.DAGHTGNGVKVAVLDTGIDCSHEDL..NVSGGHSVFTDSANN
WP_146817050.1                       SQTVPWGVPHVQGTTAQ.DAGYTGNGVKVAILDTGIDASHEDL..NVVGGYSVFDDSANN
WP_188208160.1                       SQTEPWGIPHVEGTDSR.DEGYTGEGVNVAVLDTGIDASHEDL..DVAGGHSVFDDAENS
WP_146817048.1                       SQTEPWGVPHVQGNDAQ.EEGHTGEGVSVAVLDTGIDNTHEDL..NVAGGYSVFDDAENS
WP_035661169.1                       AESVPWGVPHVQGTTAQ.ANGFTGSGVKVAILDTGIDLSHEDLSANVKGGFSVFDDAANR
WP_027965007.1                       NQTTPYGIDQVQATEAQ.NQGHTGEGVDVAVLDTGIDASHEDLAANVQGGHSVFDDAENS
WP_202078324.1                       GQTVPWGVPHVQGTAAH.EGGHTGSGVKVAILDTGIDNEHEDLAANVKGGYSVFTDDENS
WP_129080804.1                       GQTVPWGVPRVQSPEAN.NLGFTGKGIKVAILDTGIDRNHEDLSANVKGGFSVFTDTANS
WP_100374144.1                       GQAVPWGVPHVQGTAGH.DLGFTGNGVKVAILDTGIDNTHEDLAANVKGGHSVFTDAANL
WP_110612024.1                       GQTVPWGVPHVQGTAAQ.EEGYTGDSVKVAILDTGVDNTHEDLAANVKGGHSVFDDADNS
WP_230895209.1                       GQTVPWGVPHVQGTAAQ.EQGHTGDNVKVAILDTGVDNTHEDLADNVKGGHSVFDDSANS
WP_075683870.1                       AQNVPWGIPRVQGTDAQ.NAGYTGNGVKVAILDTGIDRSHPDLTANVQGGHSVFTDSANR
WP_047973355.1                       AQTTPWGIPRVQGTAAQ.DAGFTGNGVKVAILDTGIDRNHPDLSANVKGGHSVFTDSANS
WP_138811387.1                       GQTVPWGIPHVQGTDAQ.DAGYTGAGLKVAILDTGIEATHEDLADNVKGGYSVFTDADNS
WP_122897711.1                       GQTVPWGVPHVQGTAAQ.DAGYTGAGLKVAILDTGIDRNHEDLAANVKGGHSVFTDSANN
WP_101331250.1                       GETVPYGIDQVQGTQAQ.NSGFTGSGVDVAVLDTGIDRSHEDLTANVQGGYSVFDDAENS
WP_216831833.1                       GQTVPWGVPHVQSTEVH.NMGQTGAGVKVAILDTGIDNTHEDLAVNVKGGYSVFTDSANR
WP_226516443.1                       GQTVPWGVPHVQGTDVH.DMGYTGSGLNVAILDTGIDNTHEDLAANVKGGYSVFDDADNS
WP_144089130.1                       GQTVPYGIPQVEGTTAQ.DNGFSGDGVKVAVLDTGIDRSHSDL..NVAGGFSAYDSGANA
WP_096188791.1                       SQTTPWGITRVQAPDAH.QMGYSGNGVKVAILDTGIDASHPDLQANVQGGYSVFSDSANN
WP_202080138.1                       TQTVPWGVPRVQGTDAQ.NAGYTGSGVQVAILDTGIDRSHEDL..TVYDGYSVFTDSANR
WP_167261846.1                       AQDTPWGVPHVEGDTSQ.NNGYTGAGVDVAVLDTGIDRSHEDL..NVSSGHSVFGD....
WP_027963976.1                       AQQTPWGIPRVEGTTSQ.NNGYTGDGIDVAVLDTGIDRSHVDL..NVSGGYSVFGD....
WP_091776386.1                       AQTVPWGVPHVQGTTSQ.NNGYSGSGVKVAVLDTGIDSSHVDL..NVAGGYSVFGD....
WP_134338579.1                       AQQTPWGIPHVQGTQSQ.NSGYTGSGVKVAVLDTGIDSSHEDL..NVSGGYSVFGD....
WP_101332746.1                       AQETPWGIPHVEGTEAQ.NNGYTGSGVDVAVLDSGIDSSHEDL..NVAGGHSVFGD....
WP_163537364.1                       GQETPWGVPHVKATEVQ.ESGITGTGVKVGVLDTGVDFLHEDL..NVSGGATFVSD..TD
WP_099092793.1                       GQEVPWGYTHINADDVQ.GLGGTGYGVNIGVMDTGIDNFHEDL..NVVGGETFVDG..TS
WP_164853199.1                       GQEVPWGVPHIHADDVN.QLGGTGSGVNIAVMDSGIDYTHQDL..NVVGGATFVNG..TL
WP_090774843.1                       SQTVPDNIEQVKAPVAH.QRGLTGAGVKVAVLDTGIA.PHPDL..HIAGGVSFVSG..E.
WP_090775603.1                       RQTTPENIMQIGAPAAH.RRDLSGEGVKVAVLDTGIA.NHPDL..RIAGGVSFIDG..E.
WP_059104808.1                       EQAIPPGVENVGALVSH.YRGELGAGADVAIIDSGIA.AHEDL..NVVGGVSFVDS..EP
WP_035392836.1                       AQSIPWGIERIGTPAAH.ASGFTGSGVSVAVLDTGID.PHSDL..NVQGGVSFVPG..ES
WP_078393865.1                       AQTIPWGISQISAPEAQ.IAGFTGEGVNVAVLDTGIE.DHPDL..NVQGGVSFVQG..EP
EZH65969.1                           SQTIPWGIDRVNAPAAN.ASGVTGGGVSVAVLDTGIS.THEDL..NIQGGESFVPG..EP
KMK76635.1                           NQTVPWGIAQVQAPQAH.ELGHSGSGTKVAVLDTGIA.EHADL..FIHGGASFVAG..EP
WP_143850013.1                       QQTVPWGIQRVQAPAVI.NRGINGSGVRVAVLDSGIS.SHSDL..SISGGVSFVPG..EP
WP_203087429.1                       QQTVPWGITRVQAPAVH.NRGITGSGVRVAILDSGIS.AHSDL..NIRGGASFVPG..EP
WP_059105057.1                       QQSVPWGIQRVQAPAAN.NRGFTGAGVQVAVLDTGIA.SHSDL..NIRGGASFISG..EP
S08.098_sub._sendai                  NQVTPWGITRVQAPTAW.TRGYTGTGVRVAVLDTGIS.THPDL..NIRGGVSFVPG..EP
WP_003321226.1                       NQVTPWGITRVQAPTAW.TRGYTGTGVRVAVLDTGIS.THPDL..NIRGGVSFVPG..EP
WP_034632645.1                       NQTIPWGITRVQAPAAI.NRGFTGAGVRVAVLDTGIS.NHPDL..NIRGGVSFVPG..ES
S08.157_sub._YaB                     MQTVPWGINRVQAPIAQ.SRGFTGTGVRVAVLDTGIS.NHADL..RIRGGASFVPG..EP
WP_060704798.1                       MQTVPWGINRVQAPIAQ.SRGFTGTGVRVAVLDTGIS.NHADL..RIRGGASFVPG..EP
WP_095239263.1                       AQSVPWGISRVQAPAAH.NRGLTGSGVKVAVLDTGIS.THPDL..NIRGGASFVPG..EP
S08.010_M-pep.                       AQSVPWGISRVQAPAAH.NRGLTGSGVKVAVLDTGIS.THPDL..NIRGGASFVPG..EP
S08.038_PB92                         AQSVPWGISRVQAPAAH.NRGLTGSGVKVAVLDTGIS.THPDL..NIRGGASFVPG..EP
S08.003_Savinase                     AQSVPWGISRVQAPAAH.NRGLTGSGVKVAVLDTGIS.THPDL..NIRGGASFVPG..EP
S08.028_ALTP                         AQSTPWGVTRVQAPNVW.NRGFTGSGVRVAVLDTGIHSSHEDL..TVSGGYSVFG...DS
WP_143849870.1                       YQEVPYGIPQVNAPAVH.EAGNFGEGVSVAVIDSGIA.QHEDL..NIVGGESFVSS..EP
WP_203088820.1                       MQEIPYGIPQVNTPVVH.EAGNVGSGVSVAVIDTGIA.QHEDL..NIVGGESFVSS..EP
WP_017729072.1                       SQTVPWGIPFIYAPEVH.NEGFFGQGVKVAVLDTGIA.PHPDL..SIRGGASFVLT..ER
WP_122896828.1                       LQTTPWGIPFIYADQVH.NRGTYGQGVRVAVLDTGIA.THADL..NIRGGASFIPN..EP
WP_047973137.1                       AQTVPWGIPFIYSDEVH.RQGYYGNGVKVAVLDTGIA.THPDL..AIKGGVSFIPT..EN
S08.045_sub._ALP_1                   AQTVPWGIPYIYSDVVH.RQGYFGNGVKVAVLDTGVA.PHPDL..HIRGGVSFIST..EN
WP_022628745.1                       AQTVPWGIPYIYSDVVH.RQGYFGNGVKVAVLDTGVA.PHPDL..HIRGGVSFIPT..EN
S08.046_sub._aprM                    SQTVPWGISFINTQQAH.NRGIFGNGARVAVLDTGIA.SHPDL..RIAGGASFISS..EP
WP_053432556.1                       SQTVPWGISFINTQQAH.NRGIFGNGARVAVLDTGIA.SHPDL..RIAGGASFISS..EP
WP_210595747.1                       AQSIPWGITRVNTQAVH.STRNFGQGVKVAVLDSGIA.SHQDL..RIAGGVSFVAT..ES
WP_129077943.1                       AQTTPWGVSHIRATTVH.SWGNYGSGVRVAVLDTGIA.SHTDL..RISGGASFISS..EP
WP_100374143.1                       TQTVPWGITHIKAPTVH.SWGNRGGGVKVAILDTGVA.NHPDL..QISGGASFIGS..EP
WP_216831504.1                       SQTTPWGINHIQAPTVH.SWGNRGNGVKVAVLDTGIA.THEDL..RISGGASFIRS..EP
WP_078596166.1                       SQTIPWGINRVQAPTVH.SWGARGNGVRVAVLDTGIA.SHEDL..RISGGASFISS..EP
WP_035666680.1                       SQTVPWGINHIQAPTVH.SWGNRGNGVRVAVLDSGVA.SHEDL..RISGGRSFITS..EP
WP_078597775.1                       NQDIPWGIPHINADDVQNNYGNFGDGVSVAVLDTGIE.HHEDL..NVAGGVSFVSG..EP
WP_100832725.1                       GQTVPYGIDHINATSVQERDENTGQGVEVAILDSGIA.PHEDL..NIADGASFIDS..EP
WP_090774498.1                       AQQVPYGVTNIRAQEVQQSDGNKGAGVRVAVLDTGIQ.HHEDL..AVAGGVSFVPS..ES
WP_096186536.1                       GQEVPYGIEHINALEVQEADGNTGNGVSVAVLDTGIQ.DHEDL..NVVGGESFIDS..EP
WP_110520788.1                       AQEVPYGVEQVGALDVQQNDGNTGAGVSVAVLDTGIA.DHSDL..NVVGGASFVAG..EP
WP_122900894.1                       AQDVPYGIEHIGALDVQQNDGNTGAGVSVAVLDTGIQ.DHEDL..NVAGGVSFIDG..EP
WP_026691049.1                       AQEVPYGIEQIGAIDVQVNDGNTGNGVSVAVLDTGIA.AHEDL..NVVDGESFIAG..EP
WP_199800957.1                       SQTIPYGIKSIKAQKVH.KRGYAGQNVKVAVLDSGIDGKHEDL..HVTGGVSFVPT..ES
S08.005_endopep._Q                   AQTVPYGIPQIKAPAVH.AQGYKGANVKVAVLDTGIHAAHPDL..NAAGGASFVPS..EP
CAO03040.1                           AQTVPYGIPQIKAPAVH.AQGYKGANVKVAVLDTGIHAAHPDL..NVAGGASFVPS..EP
WP_081105403.1                       AQTVPYGIPQIKAPAVH.AQGYKGANVKVAVLDTGIHAAHPDL..NVAGGASFVPS..EP
S08.034_sub._BPN’                    AQSVPYGVSQIKAPALH.SQGYTGSNVKVAVIDSGIDSSHPDL..KVAGGASMVPS..ET
WP_003155195.1                       AQSVPYGVSQIKAPALH.SQGFTGSNVKVAVIDSGIDSSHPDL..KVAGGASMVPS..ET
WP_003327717.1                       AQSVPYGISQIKAPAVH.SQGYTGSNVKVAVIDSGIDSSHPDL..KVSGGASFVPS..EP
WP_039073463.1                       AQSVPYGISQIKAPALH.SQGYTGSNIKVAVIDSGIDSSHPDL..NVRGGASFVPS..ET
S08.002_mesentericopep.              AQSVPYGISQIKAPALH.SQGYTGSNVKVAVIDSGIDSSHPDL..NVRGGASFVPS..ET
S08.042_amylosacchariticus           AQSVPYGISQIKAPALH.SQGYTGSNVKVAVIDSGIDSSHPDL..NVRGGASFVPS..ET
S08.035_sub._J                       AQSVPYGISQIKAPALH.SQGYTGSNVKVAVIDSGIDSSHPDL..NVRGGASFVPS..ET
S08.036_sub._E                       AQSVPYGISQIKAPALH.SQGYTGSNVKVAVIDSGIDSSHPDL..NVRGGASFVPS..ET
S08.044_sub._NAT                     AQSVPYGISQIKAPALH.SQGYTGSNVKVAVIDSGIDSSHPDL..NVRGGASFVPS..ET
WP_053604255.1                       AQTVPYGIPHIKADRVQ.AQGYSGGGVKVAVLDTGIDASHEDL..NVVGGASFVPS..EP
NPC92104.1                           AQTVPYGISLIKADKVQ.AQDVTGKNVKVGIIDTGISSAHPDL..KVAGGKSFVAG..DS
```

```
S08.001_sub._Carlsberg    AQTVPYGIPLIKADKVQ.AQGFKGANVKVAVLDTGIQASHPDL..NVVGGASFVAG..EA
AAS86761.1                AQTVPYGIPLIKADKVQ.AQGFKGANVKVAVLDTGIQASHPDL..NVVGGASFVAG..EA
S08.037_sub._DY           AQTVPYGIPLIKADKVQ.AQGYKGANVKVGIIDTGIAASHTDL..KVVGGASFVSG..ES
NUJ19608.1                AQTVPYGIPQIKADKVQ.AQGYKGANVKVGVIDTGIAASHSDL..NVVGGASFVSG..ES
WP_188377243.1            AQTTPEGIEQVNADDVQ.DSGNTGSGVKVAVLDSGIEAAHEDL..NVAGGESFISE..EP
WP_173918387.1            AQTSPPGISQINADDVQ.ATGNTGSGVKVAVLDSGIEAGHEDL..NVAGGASFVSS..EP
KGX83542.1                GQTTPWGITEINADDVQ.SNGTTGTGVKVAVLDSGISASHEDL..SVAGGASFVDG..EP
WP_051255158.1            GQTTPWGITEINADDVQ.SNGTTGTGVKVAVLDSGISASHEDL..SVAGGASFVDG..EP
WP_231417544.1            GQTTPWGIPAINADDVQ.ASGNSGSGIKVAVLDSGISASHEDL..QVAGGASFVDG..EP
WP_096155439.1            SQTVPYGVTHIKADVAH.SQGITGNGVKVAILDTGIDASHPDL..NVAGGASFVSG..EP
AST90329.1                SQTVPYGVPHIKADVAH.SQNVTGNGVKVAILDTGIDAAHEDL..RVVGGASFVAG..EP
WP_230500539.1            AQTIPYGIPHVKADVAH.SQNVTGSGVKVAVLDTGIDASHEDL..NVAGGASFVSA..EP
WP_078381234.1            AQTVPYGIPHIKADVAH.SQNVIGTGVKVAVLDTGIDSSHEDL..RVAGGASFVSG..EA
WP_060666810.1            AQSVPYGVPHIKADVAH.AQNVTGSGVKVAVLDTGIDASHEDL..RVTGGASFVSG..EP
WP_152444042.1            AQTVPYGIPHIKADVAH.AQNVTGSGVKVAVLDTGIDASHEDL..KVVGGKSFVSG..EP
WP_066412694.1            AQTTPWGVTHINAHRAH.SSGVTGSGVKVAILDTGIHASHPDL..NVRGGASFISG..ES
WP_230500606.1            AQTVPWGIPHIKANTAH.AQGVTGSGVRVAVLDTGIDANHVDL..NVRGGASFISG..ES
WP_088017821.1            AQTVPWGIPHIKADKAH.ASGVTGSGVKVAVLDTGIDANHADL..NVKGGASFVSG..EP
WP_224838688.1            AQSTPWGVTHINAQKAH.AANVTGSGVKVAVLDTGIDASHPDL..NVKGGVSFVSG..EP
WP_100334303.1            AQTTPWGITHINAHKAH.SSNITGSGVKVAVLDTGIDASHPDL..NVKGGASFVSG..EP
WP_094921089.1            GQTVPWGIPHINADDVH.ATGNTGNGVKVAVLDTGIQASHEDL..NVVGGASFIPA..EP
WP_224844257.1            SQTVPWGIPHIKADLVQ.SAGNTGSGVKVAVLDTGIQRSHSDL..TVVGGASFVPA..EP
WP_070119644.1            GQTVPWGITHINADDVQ.ATGNTGSGVKVAILDTGIDASHEDL..SVAGGASFIAA..EP
WP_181472841.1            GQTTTWGIPHIKSDQVH.ATGNTGSGVKVAILDTGIDASHEDL..NVTGGESFVSG..EP
WP_136946078.1            AQTTPWGIPHIKADQVH.ATGNTGSGVKVAVLDTGIDASHEDL..NVSGGASFVSG..EP
WP_153236691.1            AQSTPWGITHIKANQVH.ATGNTGSGVKVAILDTGIDGSHGDL..NVRGGASFVPS..EP
WP_077360649.1            AQTTPWGITHIKADQVH.ATGNTGSGVKVAILDTGIDASHADL..NVRGGASFVAG..EP
WP_206945444.1            SQTTPYGISQINADDVQ.AQGTTGNGVKVAILDSGIDGAHEDL..NVAGGESFVSG..EP
WP_029565418.1            GQSVPYGISQIKADAVQ.ASGVKGSGVKVAILDSGIDASHEDL..NVSGGASFIPN..EP
TDL80277.1                GQTVPYGIPQIKADAVQ.SSGVKGSGVKVAVLDTGIDASHEDL..NVAGGASFISS..EP
WP_224844255.1            GQTTPWGVPHIKADVVQ.STGTTGTGVKVAILDTGIDASHEDL..NVVGGASFVAA..EP
WP_193538138.1            GQTTPWGIPHIEADTVQ.ASGVTGSGVKVAILDTGIDGNHEDL..NVLGGASFISG..EP
WP_078544469.1            GQTVPWGIPHIKADTVQ.STGVTGSGVKVAILDTGIDATHEDL..NVAGGASFVSG..EP
WP_090849877.1            GQAVPWGIPHIKADTVQ.STGVTGNGVKVAILDTGIDSYHEDL..SVAGGASFVSG..EP
WP_022794977.1            AEYIPWGVDYVEAPSIQ.ETGITGQGADVAVLDTGISDNHFDL..TVTGGESFISY..EP
WP_107586282.1            AQTVPWGIDRLEAPSIH.SSGLTGSGVSVAVLDTGIEASHSDL..NVQGGESFVSG..EP
WP_106589713.1            GTPAPWGVDHLDGPDIH.NTGITGDNVDVAVLDTGIDGSHHDL..NVVGGESFVSG..EP
WP_018922084.1            NQTIPWGVDHINADNYH.G.STTGNGVDVAVLDTGIDGNHPDL..NVVDGESFVQG..EP
```

```
                               60        70        80        90        100       110
WP_105960433.1                 DPYMDGNGHGTHVAGTVAAVDNDLGVIGAAPEADLFAVKVLDNDGSGSLAGIAEGLEWSV
WP_107583584.1                 DPFYDANGHGTHVAGTIGAVDNDLGVIGAAPEADLYAVKVLSNEGSGSLAGIAEGLEWSI
WP_168006597.1                 NPFSDGNGHGTHVAGTVAAVDNDLGVLGVAPDADLHAVKVLSNDGSGSLDGIARGIEWSI
WP_090843404.1                 NPFYDENGHGTHVAGTVAALDNDIGVLGAAPEADLYAVKVLSNEGSGSLAGIAEGIEWSI
WP_147804655.1                 NPFNDGNGHGTHVAGTVAAVDNDLGVIGTAPEADLYAVKVLSNEGSGSLSGIAEGLEWSI
WP_168007760.1                 NPFFDGNGHGTHVAGTVAAVDNDLGVIGTAPDADLYAVKVLSNEGSGSLAGIAEGIEWSI
WP_146817052.1                 DPYYDGSGHGTHVAGTVAALDNNLGVLGVAPQANLYAVKVLDSNGGGSYSGIAQGIEWSI
WP_026691136.1                 SPYYDGDGHGTHVAGTVAALNNSIGVLGVAPSARLYAVKVLDSNGNGSYSGIAQGIEWSV
WP_171051829.1                 NPYYDGDGHGTHVAGTVAALNNSYGVLGVAPQARLYAVKVLDSNGGGSYSGIAQGIEWSI
WP_168009413.1                 NPYIDNDGHGTHVAGTVAALNNSVGVLGVAPQARLYAVKVLDQNGSGSNAGIAQGIEWSV
WP_124221886.1                 .PFTDENGHGTHVAGTVAAQDNDVGVVGVAPDASLYAVKVLDEDGSGSYSGIAEGIEWAV
WP_134339482.1                 .PYNDGNSHGTHVAGTVGALDNDLGVVGVAPDAELYAVKVLDDNGSGSYAGIAEGIEWAI
WP_091776380.1                 .PYDDGNGHGTHVAGTVAALDNDLGVVGVAPDADVYAVKVLDDSGSGSYAGIAEGIEWAI
WP_100334247.1                 DPYYDANGHGTHVAGTVAALNNNTGVLGVAYNAELYAVKVLNNSGSGSYAGIAQGIEWAV
S08.133_sub._LD-1              DPYYDGSGHGTHVAGTVAALNNSVGVLGVAYNAELYAVKVLNNSGSGSYAGIAEGIEWAV
WP_084380659.1                 DPYYDGSGHGTHVAGTVAALNNSVGVLGVAYNADLYAVKVLNNSGSGSYAGIAEGIEWAV
WP_134339480.1                 DPYNDGNGHGTHVAGTVAATNNTLGVLGVAPQAELYAVKVLNNDGSGSYSGIAEGIEWSI
WP_091776383.1                 DPCNDGNGHGTHVAGTVAALDNNVGVLGVAPQTDLYAVKVLSNDGSGSYSGIAEGIEWSI
WP_146817050.1                 DPFYDGSGHGTHVAGTVAALDNNVGVLGVAPQSDLYAVKVLNNSGSGSYAGIAEGLEWSI
WP_188208160.1                 DPYYDENGHGTHVAGTVAALDNDLGVVGVAPDASVYAVKVLDNSGSGSYAGIAEGIEWSI
WP_146817048.1                 DPYYDANGHGTHVAGTVAALDNDLGVIGVAPETDVYAVKVLDNNGSGSYAGIAEGIEWAI
WP_035661169.1                 DPYYDANGHGTHVAGTVAAVNNNLGVLGVAYQADLYAVKVLNNDGSGSYAGIARGIEWSV
WP_027965007.1                 DPYNDGDGHGTHVAGTVAAANNDTGVVGVAPQANLYAVKVLGNDGSGSYAGIAEGIEWSI
WP_202078324.1                 DPFYDGNGHGTHVAGTVAAVDNTLGVLGVAHQADLYAVKVLSNSGSGSYAGIAEGIEWAV
WP_129080804.1                 DPYYDGSGHGTHVAGTVAAVDNQLGVIGVAKNAELYAVKVLNNSGSGSYEGIAKGIEWSI
WP_100374144.1                 DPFYDEDGHGTHVAGTVAAINNDLGVLGVAHQADLYAVKVLNNSGSGSYAGIAAGIEWAI
WP_110612024.1                 DPYYDENGHGTHVAGTVGAVNNDLGVIGVAYDADLYAVKVLNNAGSGTLAGIAEGIEWSI
WP_230895209.1                 DPYYDANGHGTHVAGTVGAVNNDLGVIGVAYDADLYAVKVLNNDGSGTLAGIAEGIEWSI
WP_075683870.1                 DPFFDGDGHGTHVAGTVAAVNNDIGVVGVASEADLYAVKVLNNAGSGSYAGIAEGIEWSI
WP_047973355.1                 DPFFDGDGHGTHVAGTVAAVNNDIGVIGVASEASLYAVKVLNNAGSGSYAGIAEGIEWAI
WP_138811387.1                 DPYYDPNGHGTHVAGTVAAVDNDLGVIGVAPEADLYAVKVLSNAGSGSIAGIAEGIEWSI
WP_122897711.1                 DPFYDADGHGTHVAGTVAAVDNNLGVVGVATQAELYAVKVLNNNGSGSYAGIAQGIEWSI
WP_101331250.1                 DPYNDGNGHGTHVAGTVAAVDNDLGVLGVAPSTNLYAVKVLDNDGSGSYAGIAEGIEWAV
WP_216831833.1                 DPFYDGSGHGTHVAGTVGAIDNSLGVIGVAHQTDLYAVKVLNNDGSGSYAGIAEGVEWSI
WP_226516443.1                 DPFYDGNGHGTHVAGTVGAIDNDLGVLGVAHSTNLYAVKVLNNDGSGSYAGIAEGIEWSI
WP_144089130.1                 DPYNDGSGHGTHVAGTIAAQDNNLGVLGVTPNVELYAVKVLDNQGSGTYADIAEGIEWAI
WP_096188791.1                 NPFYDGDGHGTHVAGTVAAANNGGGVLGVSHQADLYAVKVLDNNGGGSYSGIAQGIEWSI
WP_202080138.1                 DPYYDGNGHGTHVAGTVAALNNHVGVLGVAPSAQLYAVKVLDNNGGGSYSGIARGIEWSI
WP_167261846.1                 SPYADGQGHGTHVAGTIGANDNNTGVVGVAPDASLHAVKVLDDDGSGSYAGIAEGIEWAI
WP_027963976.1                 SPYYDGDGHGTHVAGTIAALDNNTGVLGVAPDANLYAVKVLDNNGSGSYSGIAQGIEWSI
WP_091776386.1                 SPYNDGNGHGTHVAGTVAALNNNSGVLGVAPQASLYAVKVLDANGSGSYAGIAEGIEWAI
WP_134338579.1                 SPYNDGNGHGTHVAGTVGALDNNLGVIGVAPDASLYAVKVLDSNGSGSYSGIAQGIEWSI
WP_101332746.1                 SPYNDENGHGTHVAGTVGALDNNLGVVGVAPQANLYAVKVLDGEGSGSYSGIAEGIEWSI
WP_163537364.1                 N.YMDDNGHGTHVAGTVAAQNNTIGVIGVAPKVELYGIKVLDQSGYGSYSDVIAGIEWAT
WP_099092793.1                 D.YMDDNGHGTHVSGVISALNNNLGVLGVAAEANLYSIKVLDNNGNGYYSDVIEGIEWAM
WP_164853199.1                 S.YIDEYGHGTFVAGIVSALNNDLGVVGVASEANLYSIRVLDKYGNGNFSDVISGIEWAI
WP_090774843.1                 D.YEDENGHGTHVAGTIAALDNGIGVVGVAPEADLYAVNVLGKEGAALTSVVIEGIEWAI
WP_090775603.1                 S.YTDKNGHGTHVAGTIAARDNHIGIVGVAPDADLYAVNVLGSAGSALTSVVIQGIEWAI
WP_059104808.1                 S.YEDNNGHGTHVAGTVAALDNNHGVLGVSPAANLYAVKVLGSDGMGQNSDIIRGIDWAI
WP_035392836.1                 G.ADDGNGHGTHVAGTIAALDNDEGVLGVAPEVDLFAVKVLSASGSGSISSIAQGLEWTA
WP_078393865.1                 D.YQDGNGHGTHVAGTIAALDNDEGVIGVAPNADLYAVKVLGANGSGSVSSIAQGLEWAG
EZH65969.1                     G.IDDGNGHGTHVAGTIAALDNDTGVVGVSPDADLYAVKVLGSDGSGNISSIAQGLQWAG
KMK76635.1                     D.YHDLNGHGTHVAGTIAALNDGAGVIGVAPDAELYAVKVLGASGSGSVSSIAQGLEWAG
WP_143850013.1                 T.IADGNGHGTHVAGTIAALNNSIGVVGVAPNAQIYGVKVLGANGRGSVSGIAQGLEWAA
WP_203087429.1                 T.TADLNGHGTHVAGTVAALNNSIGVIGVAPNAELYAVKVLGANGSGSVSGIAQGLEWAA
WP_059105057.1                 S.TSDNNGHGTHVAGTIAALNNSTGVVGVAPRAELYAVKVLGADGSGSISSIARGLEWAA
S08.098_sub._sendai            S.YQDGNGHGTHVAGTIAALNNSIGVVGVAPNAELYAVKVLGANGSGSVSSIAQGLQWTA
WP_003321226.1                 S.YQDGNGHGTHVAGTIAALNNSIGVVGVAPNAELYAVKVLGANGSGSVSSIAQGLQWTA
WP_034632645.1                 T.YQDGNGHGTHVAGTIAALNNSIGVVGVAPNTELYAVKVLGANGSGSISSIAQGLQWTA
S08.157_sub._YaB               N.ISDGNGHGTQVAGTIAALNNSIGVLGVAPNVDLYGVKVLGASGSGSISGIAQGLQWAA
WP_060704798.1                 N.ISDGNGHGTHVAGTIAALNNSIGVLGVAPNVDLYGVKVLGASGSGSISGIAQGLQWAA
WP_095239263.1                 S.TQDGNGHGTHVAGTIAALNNSIGVLGVAPNAELYAVKVLGASGSGSVSSIAQGLEWAG
S08.010_M-pep.                 S.TQDGNGHGTHVAGTIAALNNSIGVLGVAPSAELYAVKVLGASGSGSVSSIAQGLEWAG
S08.038_PB92                   S.TQDGNGHGTHVAGTIAALNNSIGVLGVAPNAELYAVKVLGASGSGSVSSIAQGLEWAG
S08.003_Savinase               S.TQDGNGHGTHVAGTIAALNNSIGVLGVAPSAELYAVKVLGASGSGSVSSIAQGLEWAG
S08.028_ALTP                   P.YNDVQGHGTHVAGTIAARNNSVGVIGVAYNAQLYAVKVLNNQGSGTLAGIAQGIEWAR
WP_143849870.1                 T.YEDLNGHGTHVAGTIAALDNIIGVIGVSPDVDLYAVKVLDSFGSGYTSDIAAGIEWAA
WP_203088820.1                 S.YEDLNGHGTHVAGTVAALDNSVGVLGVAPEADLYAVKVLDQFGDGYTSDIAAGIEWAA
WP_017729072.1                 N.YTDYNGHGTHVAGTIAALDNSIGVLGVAPAAELYAVKVLNAIGSGTLSSIARGIDWSI
WP_122896828.1                 T.YQDFNGHGTHVAGTVAALNNSYGVIGVAPSAELYAVKVLDANGGGSHASIAQGIEWAV
WP_047973137.1                 T.YIDYNGHGTHVAGTVAALNNSYGVLGVAPGADLYAVKVLDRNGGGSHATIAQGIEWAM
S08.045_sub._ALP_1             T.YVDYNGHGTHVAGTVAALNNSYGVLGVAPGAELYAVKVLDRNGSGSHASIAQGIEWAM
WP_022628745.1                 T.YVDYNGHGTHVAGTVAALNNSYGVLGVAPGAELYAVKVLDRNGSGSHASIAQGIEWAM
S08.046_sub._aprM              S.YHDNNGHGTHVAGTIAALNNSIGVLGVAPSADLYAVKVLDRNGSGSLASVAQGIEWAI
WP_053432556.1                 S.YHDNNGHGTHVAGTIAALNNSIGVLGVAPSADLYAVKVLDRNGSGSLASVAQGIEWAI
WP_210595747.1                 S.YQDYNGHGTHVAGTIAGLNNNLGVLGVAPSVELYAVKVLDQSGNGSHSNIARGIEWAM
WP_129077943.1                 S.YQDYNGHGTHVAGTIAALNNSYGVIGVAPSVNLYAVKVLDRNGSGSLSGIAKGIEWAV
WP_100374143.1                 S.YQDLNGHGTHVAGTVAALNNSYGVLGVAPSSSIYAVKVLDRNGGGSHASIAQGIEWSI
WP_216831504.1                 S.YQDYNGHGTHVAGTIAALNNSYGVLGVAPSANIYAVKVLDRNGSGALSSIIQGIEWSV
WP_078596166.1                 S.YNDLNGHGTHVAGTIAARDNSYGVLGVAPNVDLYAVKVLDRNGSGSLSGIARGIEWAI
WP_035666680.1                 S.YQDYNGHGTHVAGTIAGLNNSYGVLGVAPNVNLYAVKVLDRNGSGSHSAIAQGIEWSV
WP_078597775.1                 D.YIDYNGHGTHVAGTIAALDNNYGVLGVSPDVDLYAVKVLGADGSGTISGIAQGIEWTI
WP_100832725.1                 S.YYDYNGHGTHVAGTVAALDNNVGVLGAAPDVDLYAVKVLGADGSGSFASIIQGIEWAV
WP_090774498.1                 N.YQDGNGHGTHVAGTIAALDNQIGVLGVSPDVELYAVKVLSSSGSGTLAGIARGIEWAA
WP_096186536.1                 D.YQDQNGHGTHVAGTVAALDNDLGVLGVSPDIDLYAVKVLGADGSGSHATIAQGIEWAV
WP_110520788.1                 D.YDDYNGHGTHVAGTVAALDNDFGVLGVSPDVDLYAVKVLGADGSGTMAGIAQGIEWAA
WP_122900894.1                 E.YQDENGHGTHVAGTIAALDNEVGVLGVSPDVDLYAVKVLGADGSGSHAGIVQGIEWAV
WP_026691049.1                 D.YEDYNGHGTHVAGTVAALDNDLGVLGVSPDVDLYAVKVLGADGGGSHASIAQGIEWAV
WP_199800957.1                 DPLVDPHEHGTHVAGTIAALDNKVGVVGVAPKASIYAVKVADENGDGYYSWIIKGIEWAI
S08.005_endopep._Q             NATQDFQSHGTHVAGTIAALDNTIGVLGVAPSASLYAVKALDRNGDGQYSWIISGIEWAV
CAO03040.1                     NATQDFQSHGTHVAGTIAALDNTIGVLGVAPSASLYAVKVLDRNGDGQYSWIISGIEWAV
WP_081105403.1                 NATQDFQSHGTHVAGTIAALDNTIGVLGVAPSASLYAVKVLDRNGDGQYSWIISGIEWAV
S08.034_sub._BPN'              NPFQDNNSHGTHVAGTVAALNNSIGVLGVAPSASLYAVKVLGADGSGQYSWIINGIEWAI
WP_003155195.1                 NPFQDNNSHGTHVAGTVAALNNSVGVLGVAPSASLYAVKVLGADGSGQYSWIINGIEWAI
WP_003327717.1                 NPFQDGNSHGTHVAGTVAALNNSVGVLGVAPSASLYAVKVLSSSGSGDYSWIINGIEWAI
WP_039073463.1                 NPYQDGSSHGTHVAGTVAALNNSIGVLGVAPNASLYAVKVLDSTGNGQYSWIINGIEWAI
S08.002_mesentericopep.        NPYQDGSSHGTHVAGTIAALNNSIGVLGVAPSSALYAVKVLDSTGSGQYSWIINGIEWAI
S08.042_amylosaccharitícus     NPYQDGSSHGTHVAGTIAALNNSIGVLGVSPSASLYAVKVLDSTGSGQYSWIINGIEWAI
S08.035_sub._J                 NPYQDGSSHGTHVAGTIAALNNSIGVLGVSPSASLYAVKVLDSTGSGQYSWIINGIEWAI
S08.036_sub._E                 NPYQDGSSHGTHVAGTIAALNNSIGVLGVAPSASLYAVKVLDSTGSGQYSWIINGIEWAI
S08.044_sub._NAT               NPYQDGSSHGTHVAGTIAALNNSIGVLGVAPSASLYAVKVLDSTGSGQYSWIINGIEWAI
WP_053604255.1                 SPYSDGNGHGTHVSGTVAALNNTTGVLGVAPDASLYAVKVLDSAGSGSYSGIVSGIEWAT
NPC92104.1                     DPFNDGNGHGTHVAGTVAALNNSIGVIGVAPNVSLYAIKVLDSSGSGTYSAIIDGIEWAT
```

```
S08.001_sub._Carlsberg   Y.NTDGNGHGTHVAGTVAALDNTTGVLGVAPSVSLYAVKVLNSSGSGTYSGIVSGIEWAT
AAS86761.1               Y.NTDGNGHGTHVAGTVAALDNTTGVLGVAPSVSLYAVKVLNSSGSGSYSGIVSGIEWAT
S08.037_sub._DY          Y.NTDGNGHGTHVAGTVAALDNTTGVLGVAPNVSLYAIKVLNSSGSGTYSAIVSGIEWAT
NUJ19608.1               Y.NTDGNGHGTHVAGTVAALDNSIGVLGVAPNVSLYAIKVLNSSGSGTYSAIVSGIEWAT
WP_188377243.1           DPFNDQNGHGTHVAGTVAGVDNDLGVLGVAPETDLYAVKVLDGEGSGSYSAIAEGIEWAI
WP_173918387.1           DPFDDLNGHGTHVAGTIAGVDNNVGVLGVAPDVALYAVKVLNGEGSGAYSEIAAGIEWAI
KGX83542.1               DPYNDGNGHGTHVAGTIAGLDNTLGVLGVSPDVSLYAVKVLGSDGSGTYSGIIKGVEWAV
WP_051255158.1           DPYNDGNGHGTHVAGTIAGLDNTLGVLGVSPDVSLYAVKVLGSDGSGTYSGIIKGVEWAV
WP_231417544.1           DPFNDGNGHGTHVAGTIAGVNNSLGVIGVAPSAELYAVKVLNSSGSGSYSGIAKGIEWAV
WP_096155439.1           NALTDGNGHGTHVAGTVAALNNNVGVLGIAYDVDLYAVKVLGSDGSGTLAGIAQGIEWSI
AST90329.1               NALQDGNGHGTHVAGTVAALNNQVGVLGVAYDVDLYAVKVLGADGSGTLSGIAQGIEWSI
WP_230500539.1           DALTDGNGHGTHVAGTVAALNNTVGVLGVAYDVELYAVKVLDSSGGGTLAGIAQGIEWAI
WP_078381234.1           DALTDGNGHGTHVAGTIAALNNNVGVLGVSYDVELYAVKVLSSSGSGTLSGIAQGIEWAI
WP_060666810.1           DALTDGNGHGTHVAGTIAALNNNVGVLGVSYDVNLYAVKVLGADGSGTLAGIAQGIEWAI
WP_152444042.1           DALSDGNGHGTHVAGTIAGLNNTTGVLGVAYNVDLYAVKVLGADGSGTLAGIAQGIEWAI
WP_066412694.1           NPYIDSNGHGTHVAGTVAALNNTVGVLGVAYNAELYAVKVLSASGSGTLSGIAQGVEWSI
WP_230500606.1           NPYQDGNGHGTHVAGTVAALNNSTGVLGVAYNADLYAVKVLNSSGSGTLSGIAQGIEWSI
WP_088017821.1           NALQDGNGHGTHVAGTVAALNNSTGVLGVAYNADLYAVKVLSASGSGTLSGIAQGIEWSI
WP_224838688.1           SGLTDGNGHGTHVAGTVAALNNTAGVLGVAYNADLYAVKVLSASGSGSLSGIAQGIEWAI
WP_100334303.1           NALVDTNGHGTHVAGTVAALNNTIGVVGVAYNADLYAVKVLSASGSGTLSGIAQGVEWAI
WP_094921089.1           DAFSDYNGHGTHVAGTVAGLNNNLGVLGVAPSVSLYAVKVLDGNGSGTYSGIIQGIEWAI
WP_224844257.1           DPYVDLNGHGTHVAGTIAGH...TGILGVAPSVSLYAVKVLDGNGSGTYSGIIQGIEWAI
WP_070119644.1           DPYNDGNSHGTHVAGTVAGLNNTVGVLGVAPSANLYAVKVLDSAGSGTYSGIIQGIEWAV
WP_181472841.1           DPFNDGNSHGTHVAGTVAGLDNNVGVLGVAQSANLYAVKVLDGAGSGTYSGIIQGIEWSV
WP_136946078.1           DPFTDGNSHGTHVAGTVAGLNNNVGVLGVAHTASLYAVKVLDSSGSGTYSGIIQGIEWAV
WP_153236691.1           NALVDGDGHGTHVAGTVAALNNTTGVLGVAYSADLYAVKVLDSSGSGTYSGIIQGIEWAV
WP_077360649.1           NALTDGNSHGTHVAGTVAALNNTTGVLGVAYSADLYAVKVLDSSGSGTYSGIIQGIEWAV
WP_206945444.1           NALVDGNGHGTHVAGTVAGVNNTLGVLGVAPSTELYAVKVLSSEGSGSYSGIAQGIEWAI
WP_029565418.1           DPFVDGDSHGTHVAGTVAALNNTVGVLGTAPDVSLYAVKVLDSTGSGSYSGIAQGIEWAV
TDL80277.1               NPFIDGDSHGTHVAGTVAALNNSTGVLGAAPDVSLYAVKVLDSSGSGSYSGIAQGIEWAV
WP_224844255.1           NALVDGHSHGTHVAGTVAAVNNSLGVVGVAPNVDLYAVKVLDSNGSGSLSDIAKGIEWSI
WP_193538138.1           NALEDGNGHGTHVAGTVAGLNNTLGVLGVAPAADLYAVKVLDSSGSGSFSGIVQGIEWAV
WP_078544469.1           DALTDGNGHGTHVAGTVAGLNNTLGVLGVAPSASLYAVKVLGADGSGTYAGIAQGIEWAV
WP_090849877.1           NALTDGNGHGTHVAGTVSGLNNSLGVLGVAPSASLYAVKVLGADGSGTYSGIAQGIEWAI
WP_022794977.1           SPFEDGNGHGTHVAGTIAALDNGSGLIGVANNTQLHAVKVLDSSGSGSLSTIIKGIEWSI
WP_107586282.1           DPYSDSNGHGTHVAGTVGALDNSHGVLGVAPAADLYAVKVLGAEGGGTLDGIIAGIEWSI
WP_106589713.1           DPMNDENGHGTHVAGTVAALDNGTGLLGMAPDVDLHAVKVLGADGGGTLSGIAQGIEWAI
WP_018922084.1           DPFQDDNGHGTHVAGTVAALDNNEGVLGVAPDVNLHAVKVLGGDGGGTLSGIAQGIEWSI
```

```
                              120       130       140       150       160       170
WP_105960433.1                QNDIDIINMSLGGSTGSSILEEFTDLA.YDEGALVVAAAGNSGSGYGFFNTVGYPARYDS
WP_107583584.1                DNDIDIINMSLGGSSGSSILEDFTDLA.YEEGSLVVAAAGNSGNRGGNNDTVGYPANYES
WP_168006597.1                ENDMDIINMSLGGASGSSVLENFTDLA.YDEGILVVAAAGNDGKGMGFFDTVGFPAQYES
WP_090843404.1                ANDIDIINMSLGGSQGSSILEEFTDLA.YQEGSLVVAAAGNSGKGLGFFNTVGYPAQYDS
WP_147804655.1                ENDIDIINMSLGGSQGSSILEDFTDLA.YEEGSLVVAAAGNEGNGFGFFDTVGYPAQYDS
WP_168007760.1                ENDIDIINMSLGGSSGSSVLESFTDLA.FEEGSLVIAAAGNSGNRGGNNDTVGFPAKYDS
WP_146817052.1                INNMDIINMSLGGPTHSSILQAYSDYA.YNQGILVVAAAGNSGNAWGSGDNVGYPAQYDS
WP_026691136.1                NNGMDIINMSLGGSQHSSILQAYSDYA.YNQGVLLVAAAGNSGNASGTGDSVNFPAKYNS
WP_171051829.1                NNGMDIINMSLGGTAHSSILQAYSDYA.YNQGLLVIAAAGNSGNAAGTGDSVNYPARYSS
WP_168009413.1                LNNMDIVNMSLGGPTDSAILRAYSDYA.YNQGLLLIAAAGNSGNASGTGDSVGYPAKYDS
WP_124221886.1                ENDADVINMSLGGSTDSSVLEEFVDLA.YEEGVLVVAAAGNDGNRGGNNDTVGYPAKYDS
WP_134339482.1                ENNMDIINMSLGGSTDSSVLEEFVDLA.YEEGILVVAAAGNSGTWLGWFDTVGYPAKYDS
WP_091776380.1                ENDMDIINMSLGGSTSSSVLKEYVDLA.YEEGLLVVAAAGNSGTWLGWFDTVGYPAKYDS
WP_100334247.1                QNGMDIINMSLGGSQGSSILEQWCNIA.YNSGVLVVAAAGNEGRSNGRGDTVGYPAKYDS
S08.133_sub._LD-1             NNGMDIINMSLGGSMSSSILEEWCNIA.YNSGVLVVAAAGNSGRTNGRGDTVGYPAKYDS
WP_084380659.1                NNGMDIINMSLGGSMSSSILEEWCNIA.YNSGVLVVAAAGNSGRTNGRGDTVGYPAKYDS
WP_134339480.1                QNGMDIINMSLGGSQSSSILEDFANLA.YDEGLLVVAAAGNSGNRGGKNDSVGYPAKYSS
WP_091776383.1                QNDMDVINMSLGGSQSSSILEDYTNLA.YEEGLLVVAAAGNDGNRGGNNDTVGYPAKYES
WP_146817050.1                QNDMDIVNMSLGGSQSSSILEAYTDLA.NDEGVLVVAAAGNSGNRGGNNDSVGYPAKYDS
WP_188208160.1                QNDMDIINMSLGGSQSSSILEEYTDLA.YEEGVLVVAAAGNDGNRGGNNDSVGYPAKYDS
WP_146817048.1                QNDMDIINMSLGGSQSSDILEDYTDLA.YDEGSLVVAAAGNDGNRGGNNETVGYPANYES
WP_035661169.1                QNGMDIVNMSLGGSTSSSILKEWSDLA.YAQGVLLVAAAGNSGTRPGRGDNVGYPAKYDS
WP_027965007.1                NNGMDVVNMSLGGPTSSPILEEFADLA.NEEGLLVVAAAGNSGSSLGWFDTVNYPAKYDS
WP_202078324.1                KNDMDIINMSLGGSSSSSVLEEMCNAA.YDAGVLTVAAAGNSGNRGGKGDSVGYPAKYES
WP_129080804.1                QNGMDIINMSLGGSSSSSILEQFCNLA.YQEGILVVAAAGNSGTRPGRGDNVGYPAKYSS
WP_100374144.1                QNDMDIVNMSLGGSQSSAILKEFCDLA.YAEGLLVVAAAGNEGNRGGNNDTVGYPAKYDS
WP_110612024.1                DNEMDIINMSLGGSQGSSILEEFSDLA.YDEGVLVVAAAGNSGNRGGNNDTVGYPANYDS
WP_230895209.1                ENDMDIINMSLGGSQGSSILEEFSDLA.FDEGLLVVAAAGNSGNRGGNNDTVGYPAKYDS
WP_075683870.1                NNGMDIINMSLGGSQSSAILKEFSDLA.YAEGLLVVAAAGNSGNRGGNNDTVGYPAKYES
WP_047973355.1                NNDIDIINMSLGGSQSSAILKEFSDLA.YAEGVLVVAAAGNSGTRSGRNDTVGYPAKYDS
WP_138811387.1                DNDMDIINMSLGASQGSSILEQFSNLA.YDEGLLVVAAAGNSGNRGGNNNTVGYPAAYDS
WP_122897711.1                NNGMDIINMSLGGSQSSSILEQFSNLA.YEEGLLVVAAAGNSGNRGGNNDTVGYPAKYDS
WP_101331250.1                QNDMDVINMSLGGSQSSSVLEEFSNLA.NNEGLLVVAAAGNDGNRGGNNDTVGYPAKYDS
WP_216831833.1                LNGMDIINMSLGGSQSSSILKEFCDLA.YSEGLLVVAAAGNSGNRGGNNDSVGYPAKYES
WP_226516443.1                QNDMDIINMSLGGSQSSSILEEFSNLA.YEEGVLVVAAAGNSGNRGGNNDTVGYPANYES
WP_144089130.1                QNNMDVINMSLGGSSSSSILKEYSDLA.YDSGILVVAAAGNEG.SFLWFDTVGYPAAYDS
WP_096188791.1                NNDMDIINMSLGGSAHSSILEAYSNLA.YNEGILVVAAAGNSGNAWGIGDTVAYPAKYDS
WP_202080138.1                NNNMDVINMSLGGSSSSSILKDWCDYA.YNRGILLVAAAGNSGNSSGWGDTVGYPAKYDS
WP_167261846.1                QNDMDVINMSLGGSSSSSILKEYSDMA.NSEGLLVVAAAGNSGNSWGWGDTVGYPAKYDS
WP_027963976.1                INGMDIINMSLGGPSSSSILQQYSDLA.YNNGILVVAAAGNSGNSWGWGDTVGYPAKYDS
WP_091776386.1                QNDMDIINMSLGGSQSSSILKDYSDYAYYNAGILVVAAAGNSGNSWGWGDTVGYPAKYDS
WP_134338579.1                QNDMDIINMSLGGSSSSSILEDYSNLA.YNEGILVVAAAGNSGNYWGWGDTVGYPAKYDS
WP_101332746.1                QNGMDVINMSLGGSTSSSILEEYSNLA.YDEGLLVVAAAGNSGNSWGWGDTVGYPAKYDS
WP_163537364.1                TNNLDILNMSFSGETGSRTLQRALDNA.YNSGVLLVGAAGNNGFD..RKGNVGYPAKYDS
WP_099092793.1                MNQIDILNMSFGNSSSSLALEEAIDTA.YNNGMLIVASAGNNGYS..KKGSLTYPAKYSS
WP_164853199.1                SNNIDIINMSFGSNIGSKSLKKAIDKA.YNEGILMVAAVGNDGYS..KKGNVNYPAKYKY
WP_090774843.1                ENDMDVVNLSLGGGAPSQALEETVDAA.REKGILVVAASGNAGTSS.....IDYPARYES
WP_090775603.1                ENDMDIVNLSLGSNAPSRTLEAAVDAA.RENDVLVVAASGNDGHPF.....VSYPARYLS
WP_059104808.1                ENDIDIANLSLGGPTPSQALEDAVNRA.DNSGVLLVAATGNSGAGS.....VSYPARYDS
WP_035392836.1                ENNIDVANLSLGSPSPSQTLEQAVNDA.TDSGVLVVAAAGNSGTSS.....LGYPARYDN
WP_078393865.1                ENGMDIANLSLGSSAPSATLEQAVDEA.TANGVLVVAASGNSGASS.....IGYPARYDN
EZH65969.1                    ENGMDVANMSLGSPLPSPTLEQAVDEA.TDRGVLVVAASGNSGASS.....LSYPAAYDN
KMK76635.1                    DNGMDVANLSLGSPVGSDTLEQAVNYA.TDSGVLVVAASGNSGSGT.....VSYPARYDN
WP_143850013.1                TNNMDIANLSLGSDAPSSTLEQAVNFA.TSRGVLVVAASGNNGSGN.....VGYPARYAN
WP_203087429.1                TNNMHIANMSLGSDFPSSTLERAVNYA.TSRDVLVIAATGNNGSGS.....VGYPARYAN
WP_059105057.1                NNNMDIANMSLGSASPSITLERAVNQA.TNQGVLVVAASGNSGASS.....LSYPARYQN
S08.098_sub._sendai           QNNIHVANLSLGSPVGSQTLELAVNQA.TNAGVLVVAATGNNGSGT.....VSYPARYAN
WP_003321226.1                QNNIHVANLSLGSPVGSQTLELAVNQA.TNAGVLVVAATGNNGSGT.....VSYPARYAN
WP_034632645.1                QNNIHVANLSLGSPTGSQTLELAVNQA.TSAGVLVVAASGNNGSGT.....ISYPARYAN
S08.157_sub._YaB              NNGMHIANMSLGSSAGSATMEQAVNQA.TASGVLVVAASGNSGAGN.....VGFPARYAN
WP_060704798.1                NNGMHIANMSLGSSAGSATMEQAVNQA.TASGVLVVAASGNSGAGN.....VGFPARYAN
WP_095239263.1                NNGMHVANLSLGSPSPSATLEQAVNSA.TSRGVLVVAASGNSGAGS.....ISYPARYAN
S08.010_M-pep.                NNGMHVANLSLGSPSPSATLEQAVNSA.TSRGVLVVAASGNSGAGS.....ISYPARYAN
S08.038_PB92                  NNGMHVANLSLGSPSPSATLEQAVNSA.TSRGVLVVAASGNSGAGS.....ISYPARYAN
S08.003_Savinase              NNGMHVANLSLGSPSPSATLEQAVNSA.TSRGVLVVAASGNSGAGS.....ISYPARYAN
S08.028_ALTP                  QNNMHVINMSLGGTSGSTTLQNAVNAA.YNAGILVVAAAGNSGNSAGTGDNVGFPARYPN
WP_143849870.1                DQGIDIANLSLGGPTGSTVLEQAVDYA.EEQGTLLIAAAGNSGTRG.....IGYPAAYDN
WP_203088820.1                NQDIDIANLSLGGPIGSPVLEQAVDYA.EEQGTLLIAAAGNSGTRG.....IGFPAAYDN
WP_017729072.1                SNDMDIINMSLGGTSGSDTLQEAAESA.YNSGILLVAAAGNSGESG.GTNNMGFPARYST
WP_122896828.1                SNNMDIVNMSLGSPQGSTTLQQAVNNA.HNSGVLLIAAAGNSGTNG.SQNTMGYPARYSN
WP_047973137.1                NNGMDVVNMSLGSTSGSTTLQLASDRA.SSAGVLLIGAAGNSGQQG.GSNNMGYPARYNS
S08.045_sub._ALP_1            NNGMDIANMSLGSPSGSTTLQLAADRA.RNAGVLLIGAAGNSGQQG.GSNNMGYPARYAS
WP_022628745.1                NNGMDIANMSLGSPSGSTTLQLAADRA.RNAGVLLIGAAGNSGQQG.GSNNMGYPARYAS
S08.046_sub._aprM             NNNMHIINMSLGSTSGSSTLELAVNRA.NNAGILLVGAAGNTGRQG.....VNYPARYSG
WP_053432556.1                NNNMHIINMSLGSTSGSSTLELAVNRA.NNAGILLVGAAGNTGRQG.....VNYPARYSG
WP_210595747.1                NNDMDIVNMSLGGPTGSTTLQRAADAA.YNRGVLLIAAAGNTGRSG.....VGYPARYSS
WP_129077943.1                NNNMDIVNMSLGGSSGSTALQQAADNA.YNRGVLLIAAAGNTGSNG.....IQFPARYNS
WP_100374143.1                SNGMDIVNMSLGGPTGSNTLKQAVDNA.YNLGVLLVAASGNTGTAG.....IQFPARYNT
WP_216831504.1                SQNMDIVNMSLGTSSGSTSLRQAVDNA.NNRGLLLIAAAGNTGTSG.....VMFPAAYNS
WP_078596166.1                TNNMDIVNMSLGGSTGSTALRQAADNA.YNRGILLVAAAGNTGSAG.....ISFPARYNS
WP_035666680.1                SNGMHIVNMSLGGPTGSTTLQRAADNA.YNRGVLLIAAAGNTGSAG.....ISYPARYNS
WP_078597775.1                DNDIDIANMSLGSSSGSSALEIAANNA.NNAGVLLIAAAGNSGEL.LWFNTIGYPARYDS
WP_100832725.1                DNNMDVVNMSLGASRGSNALEQAVDQA.YEHGVLLVAAAGNSGTQ.GRRDTMGYPAKYDS
WP_090774498.1                DHDMDIVNMSLGGSSGSQTLEQAVNYA.HGEGVTLVAAAGNSGSF.LWFNTIGYPAKYEN
WP_096186536.1                ENDIDVVNMSLGGSTGSNTLEEAVNYA.HSEGVTLVAAAGNSGSF.AWFNTIGYPAKYDN
WP_110520788.1                DNGMDVANMSLGADMGSTTLEQAVNYA.HSQGVTLVAAAGNSGSL.GNLNTIGYPAKYDN
WP_122900894.1                ENDIDVINMSLGAPVGSTTLEQAVNYA.HGEGVTVVAAAGNEGSLIPGWNTIGYPAKYDN
WP_026691049.1                DNDIDVVNMSLGGAVGSTTLEQAVNYA.HSQGVTLIAAAGNEGSLIPGLNTIGYPAKYDN
WP_199800957.1                ENDMDIINISMGGASESEALKEAVDRA.YDKGILIVASAGNAGSY.GSLNTIDYPAKYSS
S08.005_endopep._Q            ANNMDVINMSLGGASGSTALKNAVDTA.NSRGVVAVAAAGNSGSS.GSRSTVGYPAKYES
CAO03040.1                    ANNMDVINMSLGGPNGSTALKNAVDTA.NNRGVVVVAAAGNSGSF.GSTSTVGYPAKYDS
WP_081105403.1                ANNMDVINMSLGGPNGSTALKNAVDTA.NNRGVVVVAAAGNSGSI.GSTSTVGYPAKYDS
S08.034_sub._BPN'             ANNMDVINMSLGGPSGSAALKAAVDKA.VASGVVVVAAAGNEGTS.GSSSTVGYPGKYPS
WP_003155195.1                ANNMDVINMSLGGPSGSAALKAAVDKA.VASGVVVVAAAGNEGTS.GGSSTVGYPGKYPS
WP_003327717.1                SNNMDVINMSLGGPQGSTALKAVVDKA.VSQGIVVVAAAGNSGSS.GSTSTVGYPAKYPS
WP_039073463.1                SNKMDVINMSLGGPSGSTALKSVVDRA.VASGIVVVAAAGNEGTS.GSASTIGYPAKYPS
S08.002_mesentericopep.       SNNMDVINMSLGGPTGSTALKTVVDKA.VSSGIVVAAAAGNEGSS.GSTSTVGYPAKYPS
S08.042_amylosaccharititicus  SNNMDVINMSLGGPSGSTALKTVVDKA.VSSGIVVAAAAGNEGSS.GSSSTVGYPAKYPS
S08.035_sub._J                SNNMDVINMSLGGPSGSTALKTVVDKA.VSSGIVVAAAAGNEGSS.GSSSTVGYPAKYPS
S08.036_sub._E                SNNMDVINMSLGGPTGSTALKTVVDKA.VSSGIVVAAAAGNEGSS.GSTSTVGYPAKYPS
S08.044_sub._NAT              SNNMDVINMSLGGPTGSTALKTVVDKA.VSSGIVVAAAAGNEGSS.GSTSTVGYPAKYPS
WP_053604255.1                ANGMDVINMSLGGSSGSKALKQAVDNA.YANDVVVVAAAGNSGSSGGRVNTIGYPAKYSS
NPC92104.1                    DNHMDVINMSLGGSSGSTALKKAVDRA.YSSGVVVVAAAGNSGSSGS.RNTIGYPAKYDS
```

```
S08.001_sub._Carlsberg    TNGMDVINMSLGGPSGSTAMKQAVDNA.YARGVVVVAAAGNSGSSGN.TNTIGYPAKYDS
AAS86761.1                TNGMDVINMSLGGASGSTAMKQAVDNA.YARGVVVVAAAGNSGSSGN.TNTIGYPAKYDS
S08.037_sub._DY           QNGLDVINMSLGGPSGSTALKQAVDKA.YASGIVVVAAAGNSGSSGS.QNTIGYPAKYDS
NUJ19608.1                ANNLDVINMSLGGTSGSTALKQAVDKA.YASGVVVVAAAGNSGTSGS.SSTIGYPAKYDS
WP_188377243.1            DNDMDVINMSLGGSIGSSALEQAVNNA.DDSGVLVVAAAGNEGSF.GPFNTIGYPAKYDA
WP_173918387.1            ANDMDVINMSLGGSVGSSALKEAVDNA.YESGILVVAAAGNSGKF.GPFNTIGYPAKYDT
KGX83542.1                SNNIDVVNMSLGGSRGSTSLQQAMDNA.YNSGVLLVAAAGNDGTR.GKKNTIGYPAKYSS
WP_051255158.1            SNNIDVVNMSLGGSRGSTSLQQAMDNA.YNSGVLLVAAAGNDGTR.GKKNTIGYPAKYSS
WP_231417544.1            DNNIDVVNMSLGGSRGSTTLEQAMDQA.YQQGVLLIAAAGNEGSK.GKKNTIGYPAKYAS
WP_096155439.1            ANGMDVINMSLGGSTGSSTLKQASDNA.YNSGIVVVAAAGNSGNFFGLINTIGYPARYDS
AST90329.1                ANNMDVINMSLGGSTGSTTLKQAADNA.YNSGLVVVAAAGNSGDFFGLINTIGYPARYDS
WP_230500539.1            DNNMDVINMSLGGSIGSTTLRRASDNA.YNSGIVVVAAAGNSGSGLGLLNTIGYPARYDS
WP_078381234.1            DNEMDVINMSLGGSTGSSTLKQASDNA.YNSGIVVVAAAGNSGSFFGLINTIGYPARYDS
WP_060666810.1            DNDMDVINMSLGGSTGSTTLKQASDNA.YNSGIVVVAAAGNSGSFLGLVNTIGYPAKYDS
WP_152444042.1            DNNIDVINMSLGGSTGSTTLKQACDNA.YNSGVVVVAAAGNSGSFFGLVNTIGYPAKYDS
WP_066412694.1            ANKMDVINMSLGGSSGSTALQRAVDNA.YRNNIVVVAAAGNSGAQ.GNRNTIGYPARYSS
WP_230500606.1            SNGMHVINMSLGASSGSTALQRACDNA.YARGIVVIAAAGNSGAI.GNQNTIGYPARYSS
WP_088017821.1            ANDMDVINMSLGGSTGSTALQQACDNA.YASGIVVVAAAGNSGSR.GKRNTMGYPARYSS
WP_224838688.1            ANDMDVINMSLGGSTGSTALKQACDNA.YASGIVVVAAAGNSGTK.GKQNTIGYPARYSS
WP_100334303.1            ANNMDVINMSLGGSSGSTALQQAVDNA.YASGIVVVAAAGNSGTR.GRQNTMGYPARYSS
WP_094921089.1            DNNMDVINMSLGGDRGSTSLQIACDNA.NNSGIVVVAAAGNSGSK.GKRNTIGYPAKYAS
WP_224844257.1            QEKVNVINMSLGGSQGSTALQQACDNA.NNSGIVVVAAAGNSGSK.GKRNTIGYPAKYAS
WP_070119644.1            SNDMDVISMSLGGSRGSTSLQQAVDNA.YNSGVLVVAAAGNDGAK.GKRNTIGYPAKYAS
WP_181472841.1            DNNMDVINMSLGGRTGSAALKDAVDNA.YNSGVLVVAAAGNEGSF.LVFNTIGYPAKYDS
WP_136946078.1            DNNMDVINMSLGGSSGSTALKDANDNA.YNAGVLVVAAAGNEGTR.GKQNTIGYPAKYAS
WP_153236691.1            ANNMDVINMSLGGSSGSTALKQACDNA.YNSGVLVVAAAGNSGTK.GKQNTIGYPAKYAS
WP_077360649.1            ANNMDVINMSLGGSSGSTALQQACDNA.YNSGVLVVAAAGNSGTR.GKQNTIGYPAKYAS
WP_206945444.1            SNDMDIINMSLGGSRGSATLEQAVNNA.DAQGVLVVAAAGNEGSK.GKKNTIGYPAKYTA
WP_029565418.1            ANGMDVINMSLGGSQDSTALKQAVDLA.YSRGVVVVAAAGNSGAK.GKRNTIGYPAKYSS
TDL80277.1                DNGMDVINMSLGGSQGSTALQQAVDQA.YNKGVVVVAAAGNSGSK.GKRNTIGYPAKYSS
WP_224844255.1            DNNMDVINMSLGGSSGSTTLKQACDNA.YNSGIVVIAAAGNSGSF.FWLNTIGYPAKYSS
WP_193538138.1            ENGMDVINMSLGASSGSTTLQQACDLA.YNSGIVVVAAAGNSGSK.GKRNTIGYPAKYAS
WP_078544469.1            ENGMDVINMSLGGSQGSTALEQAVDNA.YNSGVVVVAAAGNSGSR.GKRNTIGYPAKYSS
WP_090849877.1            SNNMDVINMSLGGSQGSTALQQAVDNA.YNNGIVVVAAAGNSGSK.GKRNTIGYPAKYSS
WP_022794977.1            SNDMDIVNMSLGTASGAESLEMASDNA.EEEGIFMVGAAGNSGTDG.ANNTIGYPARYDS
WP_107586282.1            ASDMDVINMSLGTPTHSQAMETASNNA.ADAGILVIAAAGNDGTNWFGSNTINYPARYDS
WP_106589713.1            TNDMDVINMSLGGDFGSQALEEASDNA.EAAGVMNIAAAGNSGESWFGGSTIGYPAAYDS
WP_018922084.1            DNNMDVINMSLGGDFGSQALEQASNNA.DDAGIVVIAAAGNSGTDFFGGSTIAYPAQYDS
```

```
                              180       190       200       210       220       230
WP_105960433.1                AMAVAAVDENNQRASFSSTGAEVEIAAPGVGVVSTVP.GNDYAALDGTSMASPHVAGAAA
WP_107583584.1                AMAVAAVDENNDRASFSSTGPAVEISAPGVNVLSTTP.GDTYDAFNGTSMAAPHVAGAAA
WP_168006597.1                AMAVAAVDENNNTASFSSAGPAVEISAPGVNVLSTVP.GNGYDALNGTSMAAPHVAGVAA
WP_090843404.1                AIAVAAVDANNNTASFSSAGPAVEISAPGVDIRSTTP.GNNYQSFNGTSMAAPHVAGVAA
WP_147804655.1                AMAVAAVDSNNQRASFSSAGPAVEISAPGVDILSTVP.NNGYDSLNGTSMAAPHVAGVAA
WP_168007760.1                AMAVAAVDENNNRATFSSTGPAVEISAPGVDVLSTVP.GNGYASFNGTSMAAPHVAGVAA
WP_146817052.1                VMAVAAVDQNENRASFSSTGPAVEISAPGVDVLSTYP.NNNYSSLNGTSMASPHVAGVAA
WP_026691136.1                VMAVAAVDWNNNRASFSSTGPTVEIAAPGVNIQSTFP.GNTYRSLNGTSMASPHVAGVAA
WP_171051829.1                VMAVAAVDWNNNRASFSSTGPAVEISAPGVSIQSTYP.GNQYVSLNGTSMASPHVAGVAA
WP_168009413.1                VMAVGAVDANNNRASFSSTGPAVEIAAPGVSIQSTYP.NNGYRSLNGTSMAAPHVAGVAA
WP_124221886.1                AVAVAAVDQNNNRATFSSTGSAVELSAPGVSVLSTVP.GNDYDSYDGTSMASPHVAGVAA
WP_134339482.1                AMAVAAVDQNNNRASFSSTGNAVEISAPGVGVLSSVP.GNGYASYDGTSMASPHVAGVAA
WP_091776380.1                AIAVAAVDQNNSRASFSSTGDALELSAPGVSVLSTVP.GNGYDSYDGTSMASPHVAGVAA
WP_100334247.1                VIAVAAVDSNNNRASFSSTGPAVEIAAPGVAVYSTTP.GNNYASYNGTSMASPHVAGVAA
S08.133_sub._LD-1             VIAVAAVDSSNNRASFSSTGPAVEIAAPGVNILSTTP.GNSYASYNGTSMASPHVAGVAA
WP_084380659.1                VIAVAAVDSSNNRASFSSTGPAVEIAAPGVNILSTTP.GNSYASYNGTSMASPHVAGVAA
WP_134339480.1                VIAVAAVDQNNNRASFSSTGPAVELSAPGVNILSSVP.GNSYDSYNGTSMASPHVAGVAA
WP_091776383.1                AMAVAAVDENNQRATFSSTGPAVEISAPGVNILSSVP.GNNYDSYNGTSMASPHVAGVAA
WP_146817050.1                AIAVAAIDQNNNRATFSSTGPAVELSAPGVNVLSTVP.GDNYDSYNGTSMAAPHVAGVAA
WP_188208160.1                AIAVAAVDENNQRATFSSTGPDVELSAPGVDVLSSVP.GDSYDTYNGTSMASPHVAGVAA
WP_146817048.1                AMAVAAVDENNDRATFSSTGDAVEIAAPGVDVLSTVP.GDGYDSFSGTSMAAPHVAGVAA
WP_035661169.1                VIAVAAVDQNNNRATFSSTGPAVEISAPGVSILSTIP.NNGYASYNGTSMASPHVAGVAA
WP_027965007.1                VMAVGAVDENNNRPSFSSTGPAVEIAAPGVDTLSTVP.GNDYASLSGTSMASPHVAGVAA
WP_202078324.1                VIAVAAVDADNQRASFSSTGPAVELAAPGVDVLSTVP.NNGYDRYNGTSMASPHVAGVAA
WP_129080804.1                VIAVAATDQSDKRGTFSSTGPAVEISAPGVGILSTTP.NNNYASYNGTSMASPHVAAVAA
WP_100374144.1                VIAVAAVDQNNNRPTFSSTGPAVELAAPGANILSTTP.NNRYAAYNGTSMASPHVAGVAA
WP_110612024.1                VIAVAAIDENNNRASFSSSGPAVEISAPGVSVLSTIP.GNDYAAFNGTSMASPHVAGVAA
WP_230895209.1                VIAVAAVDENNSRATFSSTGPAVEIAAPGVNVLSAVP.GNDYAAFNGTSMASPHVAGVAA
WP_075683870.1                VIAVAATDQNNQRATFSSTGPAVEISAPGAGILSTTP.NNTYASFNGTSMASPHVAGVAA
WP_047973355.1                VIAVAATDQNNQRATFSSTGPAVEISAPGVGILSTTP.NNNYASFNGTSMASPXVAGVAA
WP_138811387.1                VIAVAAVDQNNNRATFSSTGPAVEISAPGVNVLSTTP.GDNYASYNGTSMASPHVAGVAA
WP_122897711.1                VIAVAAVDQNNNRATFSSTGPAVEISAPGVSVLSTTP.GNNYAAFNGTSMASPHVAGVAA
WP_101331250.1                VMAVAAVDENNNRATFSSTGPAVEISAPGVDILSTTP.GDTYSSFNGTSMASPHVAGVAA
WP_216831833.1                VIAVAAVDANNNRASFSSTGPAVELSAPGVSVLSTVP.GNRYASYNGTSMASPHVAGVAA
WP_226516443.1                VIAVAAVDENNNRATFSSTGPAVELSAPGVSVLSTVP.GDGYDSYNGTSMASPHVAGVAA
WP_144089130.1                VVAVAAVDENNQRGSFSSVGSQVELSAPGVQILSTVP.GNGYDSYNGTSMASPHVAGVAA
WP_096188791.1                VIAVAATDQNNNRASFSSHGPAVELSAPGVNVLSTVP.GNGHQSYNGTSMASPHVAGVAA
WP_202080138.1                VMAVAATDSNNNRASFSSHGPAVEISAPGVNILSTVP.GNGYSSLNGTSMASPHVAGAAA
WP_167261846.1                VIAVAAVDQNNNRASFSSHGPDVELAAPGVGVQSTVP.GNGYDSLDGTSMASPHVAGVAA
WP_027963976.1                VIAVGAVDQNNNRASFSSHGPAVELAAPGVGVLSTVP.GNGYSSLNGTSMASPHVAGVAA
WP_091776386.1                VIAVAAVDQNNTRPSFSSHGPAVELAAPGVNVLSTTP.GNNYDSFNGTSMASPHVAGVAA
WP_134338579.1                VIAVAAIDQNNNRASFSSHGPDVELSAPGVSVLSTIP.GNSYDSFNGTSMASPHVAGVAA
WP_101332746.1                VIAVAAVDQNNNRASFSSHGPDVELSAPGVSVLSTVP.GNSYDSLNGTSMASPHVAGVAA
WP_163537364.1                VIAVGAIDQQNNRADFSSVGRELEIMAPGVTIKSTIP..GGYAFSSGTSMAAPHVVGVAA
WP_099092793.1                VISVGAVDQYDNRASFSSVGKELELVAPGIYINSTVP..GGYSIFDGTSMAAPYVTGVAT
WP_164853199.1                VMGIGAIDQNNIIADFSSNGKEVDLVAPGVLVNSTYI..NGYAILNGTSMAAPYVTGVAS
WP_090774843.1                VIAVGSVDSDNKRDATSQYGDGLSLVAPGVDVLSTFL.DGEYVEASGTSMATPAVAGAAA
WP_090775603.1                VLSVGAVDENNRRASFSQYGSGLDIVAPGVDVLSTFL.DGSYVRASGTSMATPAVAGAAA
WP_059104808.1                VLAVGAVDSSNQKANFSQYGEGLDIVAPGVEIGSTYL.GNSYHSLSGTSMASPHVAGVAA
WP_035392836.1                AMAVGATDQSDSLASFSQYGEGLDLVAPGVGVESTYP.GGGYDSLSGTSMAAPHVAGAAA
WP_078393865.1                AMAVGATDQSDSLANFSQYGEGLDIVAPGVGIDSTYT.GSSYDSLSGTSMATPHVAGSAA
EZH65969.1                    AMAVGATTQNDTRASFSQYGAGLDLVAPGVGVESTYP.GGGYRSLDGTSMATPHVAGVAA
KMK76635.1                    AFAVGATDQVNNRASFSQYGTGLDIVAPGVEVESTYL.NGEYASLSGTSMATPHVAGVAA
WP_143850013.1                AMAVGATDQNNRRANFSQYGAGLDIVAPGVGVQSTYP.GNRYVSMNGTSMASPHVAGAAA
WP_203087429.1                AMAVGATDQNNRRANFSQYGTGIDIVAPGVNVQSTYP.GNRYVSMNGTSMATPHVAGAAA
WP_059105057.1                AMAVGATDRNNNRASFSQYGAGLDIVAPGVGVQSTYL.ANRYASLSGTSMATPHVAGVAA
S08.098_sub._sendai           ALAVGATDQNNNRASFSQYGTGLNIVAPGVGIQSTYP.GNRYASLSGTSMATPHVAGVAA
WP_003321226.1                ALAVGATDQNNNRASFSQYGTGLNIVAPGVGIQSTYP.GNRYASLSGTSMATPHVAGVAA
WP_034632645.1                ALAVGATDQNNNRASFSQYGTGLNIVAPGVGVQSTYP.GNRYASLSGTSMATPHVAGVAA
S08.157_sub._YaB              AMAVGATDQNNNRATFSQYGAGLDIVAPGVGVQSTVP.GNGYASFNGTSMATPHVAGVAA
WP_060704798.1                AMAVGATDQNNNRASFSQYGAGLDIVAPGVGVQSTVP.GNGYSSFNGTSMATPHVAGVAA
WP_095239263.1                AMAVGATDQNNNRASFSQYGAGLDIVAPGVNVQSTYP.GSTYASLNGTSMATPHVAGAAA
S08.010_M-pep.                AMAVGATDQNNNRASFSQYGAGLDIVAPGVNVQSTYP.GSTYASLNGTSMATPHVAGVAA
S08.038_PB92                  AMAVGATDQNNNRASFSQYGAGLDIVAPGVNVQSTYP.GSTYASLNGTSMATPHVAGAAA
S08.003_Savinase              AMAVGATDQNNNRASFSQYGAGLDIVAPGVNVQSTYP.GSTYASLNGTSMATPHVAGAAA
S08.028_ALTP                  AMAVAATTSGNVRASFSSTGPAVEIAAPGQDINSTYP.TNTYRSLNGTSMAAPHVAGVAA
WP_143849870.1                VISVAAVDSANNKANFSSYGPENDIAAPGVDVLSTYI.GGEYTELSGTSMASPHVAGVAA
WP_203088820.1                VVSVAAVDSENNKANFSSFGPENNIAAPGVDTLSTYP.GGQYAELSGTSMASPHVAGVAA
WP_017729072.1                VMAVGAVDSDSNRASFSSYGEELEIMAPGVNIHSTYL.LNGYATLSGTSMASPHVAGVAA
WP_122896828.1                VMAVGAVDSNYNRASFSSVGNELEIMAPGVSIQSTYL.SNSYRALNGTSMAAPHVAGTAA
WP_047973137.1                VMAVGAVDQNGTRASFSSYGAELEIMAPGVNINSTHL.NNNYRSLNGTSMAAPHVAGVAA
S08.045_sub._ALP_1            VMAVGAVDQNGNRANFSSYGSELEIMAPGVNINSTYL.NNGYRSLNGTSMASPHVAGVAA
WP_022628745.1                VMAVGAVDQNGNRANFSSYGSELEIMAPGVNINSTYL.NNGYRSLNGTSMASPHVAGVAA
S08.046_sub._aprM             VMAVAAVDQNGQRASFSTYGPEIEISAPGVNVNSTYT.GNRYVSLSGTSMATPHVAGVAA
WP_053432556.1                VMAVAAVDQNGQRASFSTYGPEIEISAPGVNVNSTYT.GNRYVSLSGTSMATPHVAGVAA
WP_210595747.1                VMAVAATDSNNARASFSTYGPEVEISAPGVGINSTYP.TNSYTSLNGTSMASPHVAGVAA
WP_129077943.1                VIAVGAVNSSNQRASFSTYGSQLELMAPGVNVQSTYL.NNTYSSLNGTSMAAPHVAAVAA
WP_100374143.1                VMAVGAVDSRNRLASFSTFGNEQEIVAPGVNVQSTHL.SNGYVSLNGTSMASPHVAGAAA
WP_216831504.1                VMAVGAVDSNNNRASFSTYGSQIEIVAPGVSVLSTYT.SNRYVSLNGTSMATPHVAGVAA
WP_078596166.1                VMAVGATDSNNNRASFSTFGNELEIMAPGVSVLSTYP.TNRYVSLNGTSMASPHVAGVAA
WP_035666680.1                VMAVGAVDSNNNRASFSTFGNELEIMAPGVSILSTHL.SNQYVSLNGTSMASPHVAGVAA
WP_078597775.1                VMAVAAIDSNNNRASFSSVGGELEISAPGVSVLSTYI.GNDYASLNGTSMASPHVAGAAA
WP_100832725.1                VMAVGAVDEDNQRASFSSVGNALEVMAPGVSVYSTYL.DNSYASLNGTSMASPHVAGAAA
WP_090774498.1                VIAVGAVNENNARASFSSVGDELDVMAPGVDVLSSHL.NNAYQSFSGTSMASPHVAGAAA
WP_096186536.1                VIAVGAVDENNERASFSSVGDELDVMAPGVDVNSTYL.DDSYAELNGTSMASPHVAGAAA
WP_110520788.1                VIAVGAVDQNNERASFSSVGDELDVMAPGVSVNSTYL.NDGYQALNGTSMAAPHVAGAAA
WP_122900894.1                AIAVGAVDENNDRASFSSVGNELDVMAPGVAIDSTYL.DNSYAALSGTSMAAPHVAGAAA
WP_026691049.1                VIAVGAVDSNNNRASFSSVGNELDVVAPGVSILSTYL.GNDYAALNGTSMASPHVAGAAA
WP_199800957.1                VIAVASVDQRKQRAFDSSVGEEVEVSAPGVSTLSTIP.HNEYGYKSGTSMASPHVAGAAA
S08.005_endopep._Q            TIAVANVNSNNVRNSSSSAGPELDVSAPGTSILSTVP.SSGYTSYTGTSMASPHVAGAAA
CAO03040.1                    TIAVANVNGNNVRNSSSSAGPELDVSAPGTSILSTVP.SSGYTSYTGTSMASPHVAGAAA
WP_081105403.1                TIAVANVNSNNVRNSSSSAGPELDVSAPGTSILSTVP.SSGYTSYTGTSMASPHVAGAAA
S08.034_sub._BPN'             VIAVGAVDSSNQRASFSSVGPELDVMAPGVSIQSTLP.GNKYGAYNGTSMASPHVAGAAA
WP_003155195.1                VIAVGAVNSSNQRASFSSVGSELDVMAPGVSIQSTLP.GNKYGAYNGTSMASPHVAGAAA
WP_003327717.1                VIAVGAVDSNNQRASFSSAGSELDVMAPGVSIQSTLP.GSSYGSYNGTSMASPHVAGAAA
WP_039073463.1                TIAVGAVNSSNQRGSFSSVGPELDVMAPGVSIQSTLP.GGTYGSYNGTSMATPHVAGAAA
S08.002_mesentericopep.       TIAVGAVNSANQRASFSSAGSELDVMAPGVSIQSTLP.GGTYGAYNGTSMATPHVAGAAA
S08.042_amylosacchariticus    TIAVGAVNSSNQRASFSSAGSELDVMAPGVSIQSTLP.GGTYGAYNGTSMATPHVAGAAA
S08.035_sub._J                TIAVGAVNSSNQRASFSSAGSELDVMAPGVSIQSTLP.GGTYGAYNGTSMATPHVAGAAA
S08.036_sub._E                TIAVGAVNSSNQRASFSSAGSELDVMAPGVSIQSTLP.GGTYGAYNGTSMATPHVAGAAA
S08.044_sub._NAT              TIAVGAVNSSNQRASFSSVGSELDVMAPGVSIQSTLP.GGTYGAYNGTSMATPHVAGAAA
WP_053604255.1                VIAVGAVDSNNKKAYFSSVGDELEVMAPGVSVQSTLP.GNQYTELDGTSMASPHVAGAAA
NPC92104.1                    AIAVGAVDNNSQRAYFSSVGDELEVMAPGAAVNSTYP.GNSYKSLNGTSMASPHVAGAAA
```

```
S08.001_sub._Carlsberg    VIAVGAVDSNSNRASFSSVGAELEVMAPGAGVYSTYP.TSTYATLNGTSMASPHVAGAAA
AAS86761.1                VIAVGAVDSNSNRASFSSVGAELEVMAPGAGVYSTYP.TNTYATLNGTSMASPHVAGAAA
S08.037_sub._DY           VIAVGAVDSNKNRASFSSVGAELEVMAPGVSVYSTYP.SNTYTSLNGTSMASPHVAGAAA
NUJ19608.1                VIAVGAVNSSNQRASFSSVGPELDVVAPGVSIYSTYP.SNTYATLNGTSMASPHVAGAAA
WP_188377243.1            AMAVGAVDSDNNVASFSSRGNELEVMAPGVDVLSSIP.ENSYDEFNGTSMASPHVAGVAA
WP_173918387.1            AMAVGAVDSSNKVASFSSRGNELEVMAPGVDILSSVP.ENSYDSFNGTSMASPHVAGVAA
KGX83542.1                VMAVGAVDSNLNRASFSSVGSELEVMAPGVDIYSTLP.GNSYDYYNGTSMASPHVAGAAA
WP_051255158.1            VMAVGAVDSNLNRASFSSVGSELEVMAPGVDIYSTLP.GNSYDYYNGTSMASPHVAGAAA
WP_231417544.1            VVAVGAVDESLNRASFSSVGEELEVMAPGANIYSTLP.GNTYGSYNGTSMASPHVAGAAA
WP_096155439.1            VIAVGAVDANNNRASFSSVGNELEVMAPGVNILSTLP.GNSYGSLNGTSMASPHVAGAAA
AST90329.1                VIAVGAVDSNNRRASFSSVGSQLEVMAPGVNILSTLP.GNSYGSLNGTSMASPHVAGAAA
WP_230500539.1            VIAVGAVDSNNNRASFSSVGNQLEVMAPGVAINSTLP.GNQYGEFNGTSMASPHVAGAAA
WP_078381234.1            VIAVGAVDANNNRASFSSVGNELELMAPGVNINSTLP.GNQYGSLNGTSMASPHVAGAAA
WP_060666810.1            VIAVGAVDSNNNRASFSSVGNQLEVMAPGVSINSTLP.GNKYGELNGTSMASPHVAGAAA
WP_152444042.1            VIAVGAVDANNKRASFSSVGNELEVMAPGVSINSTLP.GNQYGELNGTSMASPHVAGAAA
WP_066412694.1            VIAVGAVDSNNNRASFSSVGSELEVMAPGVSILSTVP.GSSYASYNGTSMASPHVAGAAA
WP_230500606.1            VIAVGAVSSNNTRASFSSVGNELEVMAPGVSILSTTP.GNNYASFNGTSMAAPHVAGAAA
WP_088017821.1            VIAVGAVDSSNNRASFSSVGSELEVMAPGVSILSTTP.GNNYSSFNGTSMASPHVAGAAA
WP_224838688.1            VIAVGAVDSNNNRASFSSVGAELEVMAPGVSILSTTP.GNSYSSFNGTSMASPHVAGAAA
WP_100334303.1            VIAVGAVDSNNNRASFSSVGAELEVMAPGVSVLSTVP.GGGYASYNGTSMASPHVAGAAA
WP_094921089.1            VIAVGAVDSSNNRASFSSVGNELEVMAPGVSVYSSVP..GGYDTYNGTSMASPHVAGAAA
WP_224844257.1            VIAVGAVDSNNNRGSFSSVGNELEVMAPGVAIYSTVPYGNGYDTYNGTSMASPHVAGAAA
WP_070119644.1            AMAVGAVDDSNNRASFSSVGDELEIMAPGVNVLSSVP.GNAYDYFNGTSMATPHVSGAAA
WP_181472841.1            AMAVGAVDSNNNRASFSSVGSELEVMAPGVDILSSVP.GNSYASYNGTSMASPHVAGAAA
WP_136946078.1            VMAVGAVDSSNNRASFSSVGSELEVMAPGVSILSSVP.GNSYSSYNGTSMASPHVAGAAA
WP_153236691.1            VMAVGAVDSNNARASFSSVGSELEVMAPGVNILSSVP.GNNYASYNGTSMASPHVAGAAA
WP_077360649.1            VMAVGAVDSSNNRASFSSVGSELEVMAPGVNILSTVP.GNGYDSYNGTSMASPHVAGAAA
WP_206945444.1            AMAVGAVDSANNRASFSSVGEELEVMAPGVDILSSVP.GNNYDRYNGTSMASPHVAGAAA
WP_029565418.1            AIAVGAVDSANARASFSSVGSELEVMAPGVNILSSVP.GNKYASFNGTSMASPHVAGAAA
TDL80277.1                VIAVGAVDAANSRASFSSVGSELEVMAPGVSILSTVP.GNKYASFNGTSMASPHVAGAAA
WP_224844255.1            VIAVGAVDSTNKRASFSSVGNELEVMAPGVSILSTVP.GNRYDTFNGTSMASPHVAGAAA
WP_193538138.1            VIAVGAVDSSNNRASFSSVGNELEVMAPGVNILSSVP.GNSYDSYNGTSMASPHVAGAAA
WP_078544469.1            VIAVGAVDSSNNRASFSSVGSELEVMAPGVNILSSVP.GNGYDSYNGTSMASPHVAGAAA
WP_090849877.1            VIAVGAVDNTNNRASFSSVGNELEVMAPGVSILSSVP.GNSYDSYNGTSMASPHVAGAAA
WP_022794977.1            VMAIGSIDRYEDRSSFSSVGEELEVMAPGSSITSTYP.GQNYATLSGTSMAAPHVSGAAA
WP_107586282.1            VMAVGALDSNDNRASFSSVGNELEVMAPGADINSTYP.GNSYASLNGTSMAAPHAAGAAA
WP_106589713.1            VMAVGATDENDQRASFSSVGDSLEIMAPGVNINSTYP.GNTYESLNGTSMASPHVAGAAA
WP_018922084.1            VMAVGAVDQNNNRASFSSVGNTLEVMAPGVNIESTMP.GNNYASLDGTSMAAPHVAGTAA
```

```
                                     240       250       260       270       280
                                       .         .         .         .         .
WP_105960433.1                    QVWQAKPELTNEELRALLNETAETLDS.TYYTGNGLIQVQDAIDW...............
WP_107583584.1                    QVLEAKPDLGSSELRSLLNDTAQGLGA.SHQYGNGLVQTLDAVKK...............
WP_168006597.1                    QVWQAKPHLSNVQLRQLLNDTAEPLGA.QRDYGNGLIQSVDAINQ...............
WP_090843404.1                    QVWQARPELSNSELRELLNSTALPLGS.ARDYGNGLIQAADAVNN...............
WP_147804655.1                    QVWQSKPELSNAELRSLLNDTAAPLGA.QRDYGNGLIQTLDAIND...............
WP_168007760.1                    QVWQAKPELSSAEVRDLLNDTAVPLGA.SHQYGSGLVQSLDAIND...............
WP_146817052.1                    LVWHYRPHYSNAQLRSVLNQTAKPLGS.GNHYGNGLIQAYDAMVH...............
WP_026691136.1                    LLMHYQPGYTNAQVRSVLNNSAKPLGN.SNHFGNGLVQAFDAFVY...............
WP_171051829.1                    LLWHYQPGYSNAQIRNVLNQSAQPLGN.SNHYGNGLVQAFDAFVY...............
WP_168009413.1                    LLMHYRPNYSAQQVRSVLNNSAQPLGN.SNHFGNGLVGAYRAFVY...............
WP_124221886.1                    QVWAEKPDLSNVELRNLLQDTAQNLGD.ANKYGHGLVQSYEAINQ...............
WP_134339482.1                    QVWAAKPSLTNVELRQLLNQTAQYLGD.PFHYGNGLVQSYDAINY...............
WP_091776380.1                    QVWAEKPHLSNEELRELLNNTALDLGD.PFHYGNGLVQAVEAINY...............
WP_100334247.1                    LVWAANPGLSNVELRERLNATALNLGD.ANHFGNGLVRAMNAINGNGTTDPGNGDDDGGN
S08.133_sub._LD-1                 LVLAANPNLSNVELRNRLNDTAQNLGD.ANHFGNGLVRAVDAIN.................
WP_084380659.1                    LVLAANPNLSNVELRNRLNDTAQNLGD.ANHFGNGLVRAVDAINGTSSGDNGGGDDGG..
WP_134339480.1                    QVWGAKSGLTNVELRQLLNDTAQPLGG.SNQYGNGLVQSLDAINY...............
WP_091776383.1                    QVWAAKPDLTNVELRNLLNDTAQPLGD.SNKYGSGLVQSLDAINQ...............
WP_146817050.1                    QVWDAKPHLSNTELRELLQQTAQNLGP.SHRYGHGLVQSFEAISH...............
WP_188208160.1                    QVWAEKPELSNVELRELLQDTADELGA.SHQYGHGLVQSYEAIQH...............
WP_146817048.1                    QVWQTKPELTNEELRSLLNDTALDLGP.AHQYGHGLVQSLDAIQE...............
WP_035661169.1                    LLLENNSNLTNTELRELLQSSAKSLGT.ASQYGYGLVQAMDAINQ...............
WP_027965007.1                    SVWAEKSDLSNDELRQLLKDTAVDLGN.EDHYGAGLVQVLDALNQ...............
WP_202078324.1                    LVWGAKQGLSNEQLRQLLRDSAMDLGD.PNHYGYGLVQAVDAIQQ...............
WP_129080804.1                    LVWEAKPNLTNVELRQLLNQTAKNLGS.ATQYGHGLVQASAAIQY...............
WP_100374144.1                    QILEAKPTLSNVEVRELLRLSAKDLGN.SRQFGYGLVQTVDAIQY...............
WP_110612024.1                    QVWQAKPELSNEELRDLLNETAQSLGS.ASQYGHGLVQSLDAIEY...............
WP_230895209.1                    QVWQAKPELSNAELRELLNETAKGLGP.AHQYGHGLVQSLDAIGN...............
WP_075683870.1                    QVWQAKPHLSNVELRNLLNDTALPLGG.SNQFGNGLVQSMAAIQQ...............
WP_047973355.1                    QVWQAKPHLSNVALRNLLNETAINLGS.STQYGNGLVQSLDAIQQ...............
WP_138811387.1                    QVWQAKPGLSNTELRQLLNDTAVNLGP.AHQYGHGLVQSLDAINQ...............
WP_122897711.1                    QVWQAKPGLTNVELRNLLNDTAVNLGG.SNQYGHGLVQSLDAIQQ...............
WP_101331250.1                    LVWEAKSNLTNDELRQLLKDTAVELGA.SHQYGAGLVQAADAINQ...............
WP_216831833.1                    LIWGEHTNLTNSELRQLLQHTSINLGR.SNQYGYGLVQANDAINYLR............
WP_226516443.1                    LVWEANPGLSNVELRELLQETAVDLGA.SHQYGHGLVQAKAAIQ................
WP_144089130.1                    QVWQAKPGLSNVQLRQLLRDTAEPLGA.QREYGYGLVQSMDAINQ...............
WP_096188791.1                    LTWQAKPYLSNSQLRQTLQNTALNLGN.SNFYGHGLVRSVNAIQN...............
WP_202080138.1                    SVWQAKPWLSNAQLRQLLNNTAQYLGN.SHLYGHGLVQTYDAIRR...............
WP_167261846.1                    QVWEAKPNLTNDELRSLLQTTANDLGN.PDYYGNGLVQSYDAITQ...............
WP_027963976.1                    QVWEAKPHLSNVQLRSLLQQTAQNLGN.SNYYGSGLVKSYQAITH...............
WP_091776386.1                    QVWQAKPNLSNSQLRSLLNNTAQYLGN.SNYYGNGLVQSYNAIAN...............
WP_134338579.1                    QVWQAKPHLSNVQLRSLLQQTAQYLGD.PYYYGHGLVQSYDAIAN...............
WP_101332746.1                    QVWEAKPGLSNVELRSLLQQTAEYLGN.SDYYGYGLVQSYQAISQ...............
WP_163537364.1                    LLSESSPELDNIQVRNRLNDTATNLGD.SFYFGNGLVDAQAAISTLETTSTKGNGT....
WP_099092793.1                    LLMQLMPELTNIEIRRILNDTARPLGE.SFSYGNGLVDGQNAINFTSTNANNTKAK....
WP_164853199.1                    LLMQLNPELSNIAIEEILNQSAEPLGD.TDLYGNGLINALNAYQYSQNNVNKFK......
WP_090774843.1                    LLKEQYPHWTANEIEQRLLAETTAIGP.TFEYGQGLLNDLATK.................
WP_090775603.1                    LLKEKHPAWTASQIEMGLLTEADSLRS.RFEYGYGLLNADRATR................
WP_059104808.1                    LLKGQHPWLSNDEIRERLKETATPIGD.ALYYGSGLVNADAATHY...............
WP_035392836.1                    LVKQKNPGWTNEQIRSHLNDTANDLGD.SFRFGSGLLNAENAVQ................
WP_078393865.1                    LVKEKNPLWSNEQIRAHLNETATDLGD.TYRFGNGLLNAHAAVE................
EZH65969.1                        LVLEQNPSWSPQQVRSHINDTATDLGN.PTQFGSGLVDALSATE................
KMK76635.1                        LIKAKNPMLSNEEIRQQLVQTATPLGS.ADMYGSGLVNAEVAVQ................
WP_143850013.1                    LVKQRYPSWSNTQIRNHLKNTATNLGN.TNQFGSGLVNADAATR................
WP_203087429.1                    LVKQRYPSWNATQIRNHLKNTATNLGN.SSQFGSGLVNAEAATR................
WP_059105057.1                    LVKQKNPSWSNSQIRNHLNSTATNVGN.AQFYGNGLVNADAATR................
S08.098_sub._sendai               LVKQKNPSWSNTQIRQHLTSTATSLGN.SNQFGSGLVNAEAATR................
WP_003321226.1                    LVKQKNPSWSNTQIRQHLTSTATSLGN.SNQFGSGLVNAEAATR................
WP_034632645.1                    LVKQKNPGWSNTQIRQHLLNTATPLGS.SNQYGSGLVNAEAATR................
S08.157_sub._YaB                  LVKQKNPSWSNVQIRNHLKNTATNLGN.TTQFGSGLVNAEAATR................
WP_060704798.1                    LVKQKNPSWSNVQIRNHLKNTATNLGN.TNQFGSGLVNAEAATR................
WP_095239263.1                    LVKQKNPSWSNVQIRNHLKNTATSLGS.TNLYGSGLVNAEAATR................
S08.010_M-pep.                    LVKQKNPSWSNVQIRNHLKNTATGLGN.TNLYGSGLVNAEAATR................
S08.038_PB92                      LVKQKNPSWSNVQIRNHLKNTATSLGS.TNLYGSGLVNAEAATR................
S08.003_Savinase                  LVKQKNPSWSNVQIRNHLKNTATSLGS.TNLYGSGLVNAEAATR................
S08.028_ALTP                      LLKSARPAVTAAGIRNAMNSTALNLGN.SNWYGNGLVRANNALD................
WP_143849870.1                    LVQATNPLATAEQIGQILQETATPLGN.EEFFGSGLVDAEAAVGQ...............
WP_203088820.1                    LVQAANPSATATDIRDALQTTATPLGN.QEYFGSGLVNAEAAVGR...............
WP_017729072.1                    LIKNKHPDLSNTQIRQRINSTATYLGD.SNYYGNGLVNAEKAAQ................
WP_122896828.1                    LVKQRYPHLTNSQIRNRLNQTAIPLGN.SFYFGNGLVDAENAAGYLN.............
WP_047973137.1                    LIKQKHPHLTASQIRNRMNQTAINLGN.RTYYGNGLVDAEYAAQ................
S08.045_sub._ALP_1                LVKQKHPHLTAAQIRNRMNQTAIPLGN.STYYGNGLVDAEYAAQ................
WP_022628745.1                    LVKQKHPHLTAAQIRNRMNQTAIPLGN.STYYGNGLVDAEYAAQ................
S08.046_sub._aprM                 LVKSRYPSYTNNQIRQRINQTATYLGS.PSLYGNGLVHAGRATQ................
WP_053432556.1                    LVKSRYPSYTNNQIRQRINQTATYLGS.PSLYGNGLVHAGRATQ................
WP_210595747.1                    LVKAKYPSATNAQIRQHLRNTSTYLGS.STYYGSGLVNAQSAVN................
WP_129077943.1                    LVKSEYPWASNVQIRQRLRDTATNLGS.STYFGYGLVDALRAAY................
WP_100374143.1                    LVKSEYPWATNAQIRQRLNDTTTPLGN.AYYFGNGLVDASRAAY................
WP_216831504.1                    LVKAQYPWASNGQIRQRLRDTATPLGN.SNFYGSGVVNAYRAAY................
WP_078596166.1                    LVKSRYPNATNVQIRNRLNSTATNLGS.SYYFGNGLVNAARAAN................
WP_035666680.1                    LVKAQYPSATNAQIRQRLRDTATPLGS.SYYFGNGLVHAARAAN................
WP_078597775.1                    LVKAANPSLSNEQIRQVLNNTASPLGD.SWYYGNGLVDVDAAVRSVQ.............
WP_100832725.1                    LIIAHDPSYSHSDVRALLNDTATPLGSDSFFYGNGVIDVLAAIDSQ..............
WP_090774498.1                    LLLADDPSLSNEDIRGLLRDTAIPLGS.HFYYGKGVIDVRAAIDAN...............
WP_096186536.1                    LLLADSPNLSNEDIRQAFNETAVPLGD.HFYYGNGVIDVRAAIDGQ...............
WP_110520788.1                    LMLAEHPHLSNEDVRNVFNSTAQPLGD.HFYYGNGALDVRAALDAQ...............
WP_122900894.1                    LLLAANPSLSNDDVRAVLNETAVPLGD.HFYYGNGVIDVRAAVDAQ...............
WP_026691049.1                    LLLAENPGLTNDQVRAVFNETAVPLGD.HFYYGNGLIDVRAAIDAQ...............
WP_199800957.1                    VILSKHPNLTNDEVRDRLSKTAAQLGD.PFYYGAGLVNVQKAAR................
S08.005_endopep._Q                LILSKNPNLTNSQVRQRLENTATPLGD.SFYYGKGLINVQAASN................
CAO03040.1                        LILSKYPNLSTSQVRQRLENTATPLGD.SFYYGKGLINVQAASN................
WP_081105403.1                    LILSKYPNLSTSQVRQRLENTATPLGN.SFYYGKGLINVQAASN................
S08.034_sub._BPN’                 LILSKHPNWTNTQVRSSLENTTTKLGD.SFYYGKGLINVQAAAQ................
WP_003155195.1                    LILSKHPNWTNTQVRSSLENTTTKLGD.AFYYGKGLINVQAAAQ................
WP_003327717.1                    LVLSKHPNWTNSQVRNSLESTATNLGN.SFYYGKGLINVQAAAQ................
WP_039073463.1                    LILSKHPTWTNTQVRNRLESTTTYLGS.SFYYGKGLINVQAAAQ................
S08.002_mesentericopep.           LILSKHPTWTNAQVRDRLESTATYLGS.SFYYGKGLINVQAAAQ................
S08.042_amylosaccharitiucs        LILSKHPTWTNAQVRDRLESTATYLGN.SFYYGKGLINVQAAAQ................
S08.035_sub._J                    LILSKHPTWTNAQVRDRLESTATYLGN.SFYYGKGLINVQAAAQ................
S08.036_sub._E                    LILSKHPTWTNAQVRDRLESTATYLGN.SFYYGKGLINVQAAAQ................
S08.044_sub._NAT                  LILSKHPTWTNAQVRDRLESTATYLGN.SFYYGKGLINVQAAAQ................
WP_053604255.1                    LIKSKHPDLSASQIRQRLSDTADYLGD.HFYYGNGVINVEAAAN................
NPC92104.1                        LVKSKHPSLSASQIRDRLSKTATHLGS.AFYYGKGLINAEAAAQ................
```

```
S08.001_sub._Carlsberg   LILSKHPNLSASQVRNRLSSTATYLGS.SFYYGKGLINVEAAAQ................
AAS86761.1               LILSKHPNLSASQVRNRLSSTATYLGS.SFYYGKGLINVEAAAQ................
S08.037_sub._DY          LILSKYPTLSASQVRNRLSSTATNLGD.SFYYGKGLINVEAAAQ................
NUJ19608.1               LILSKYPTLSASQVRDRLSSTATNLGD.SFYYGKGLINVEAAAQ................
WP_188377243.1           LVFADDSSLSNDEVRTILNETATPLGD.SFDYGNGLVNAEAAVQ................
WP_173918387.1           LVMADDSSLSNQEVRNLLNETATPLGD.SFDYGNGLVNAEAAVQ................
KGX83542.1               LILANDGSLTNAEVRSLLNDTATPLGD.SFYYGNGFINVQEAVNTATTTYQVAN......
WP_051255158.1           LILANDGSLTNAEVRSLLNDTATPLGD.SFYYGNGFINVQEAVNTATTTYQVAN......
WP_231417544.1           LILAEDSSLTNQEVRSLLNSTTTPLGD.AFYYGNGLINVQEAVNAATTTFAVAQ......
WP_096155439.1           LMLAKNPNLTNVQVRQKLSQTATNLGS.KFYFGNGVINVEKALQ................
AST90329.1               LLLAQDPTLTNVQVREKLRDTATNLGS.SFYYGNGVIDVEKALQ................
WP_230500539.1           LLIAQNPNLTNVQVRERLRSTATGLGS.SWYYGNGVINIEAALQ................
WP_078381234.1           LILAKYPNLTNVEVRQKLRETATNLGS.AFNYGHGVINVEEALN................
WP_060666810.1           LLLAQNPNLTNVEVRERLRDTATNLGS.SFNYGYGVINVEAALQ................
WP_152444042.1           ILLSQNPNLTNVQVRERLRDTATNLGS.AFNYGNGVINLEAALQ................
WP_066412694.1           LLKAKYPNWSAAQIRNKLNSTTTYLGS.SFYYGNGVINVERALQ................
WP_230500606.1           LIKAKYPNMTNVQIRDRLRNTATYLGT.PFYYGNGVINVERALQ................
WP_088017821.1           LIKAKYPSMTNVQIREKLKNTATNLGD.SFYYGHGVINVESALQ................
WP_224838688.1           LIKAKYPSLSASQIRDRLKNTTTPLGD.AFYYGKGVINVEKALQ................
WP_100334303.1           LIKAKYPNLSASQIRDRLKNTATYLGD.PFYYGNGVINVEKALQ................
WP_094921089.1           LIISSNPSLSNSQVRDRLSNTATPLGS.SFYYGNGVINVQAAVQ................
WP_224844257.1           LILASNPSLSNVQVRDRLKNTATPLGS.SFYYGYGVINVQAAVQ................
WP_070119644.1           LILADNPSLSNVQVRGILNSTAIPLGD.VFYYGNGVLDVQAAVQ................
WP_181472841.1           LILAENPNLSNAEVRQKLNDTAKPLGD.SFYYGNGVIDVLEATR................
WP_136946078.1           LIIAGNSGLSNADVRQKLNDTAIPLGD.SFYYGNGVIDVYEATR................
WP_153236691.1           LIIAGNPGLTNVQVRQKLVNTAKPLGD.AFYYGKGVIDVYAATR................
WP_077360649.1           LIMAGNPGLSNVQVRQKLVNTAKPLGD.SFYYGKGVIDVYAATR................
WP_206945444.1           LILADDPTLTNDQIRQTLIDTAVPLGD.SFYYGNGVIDVQAAVQ................
WP_029565418.1           LILSKYPSMSNTEVRSRLKSTALPLGD.PFYYGAGLINVQAAIQ................
TDL80277.1               LILSKYPNMSNIEVRNRLKNTAVRLGD.PFYYGAGLINVQAAIQ................
WP_224844255.1           LILAKHPTLTNVQVRDRLKNTATYLGD.PFYYGNGVINVEKAIK................
WP_193538138.1           LILAKYPSLTNVEVRDRLKNTATPLGD.SFYYGNGVINVLAAIQ................
WP_078544469.1           LILAKHPSLTNVQVRERLRNTATYLGD.SFYYGSGVINVEAAIK................
WP_090849877.1           LILAKYPTLSNVQIRERLKNTAVPLGD.SFYYGNGVIDVEAAIQ................
WP_022794977.1           LMLDQNPSLSNEEIRLQLNNTADPAGD.SFYYGNGIIDLPEAIESE..............
WP_107586282.1           LMMDNNPSLSSEDVRSTLNETADDLGS.TFYYGNGVINLPEALYQ...............
WP_106589713.1           LMLDDDPSLSNDDIRNQLNDNAEPLGS.AFYYGNGLVDLEGILEY...............
WP_018922084.1           LMLEQNPFISNNAVRTELNDTAIDLGD.SFYYGNGLVNVDALLSE...............
```

WP_105960433.1	....
WP_107583584.1	....
WP_168006597.1	....
WP_090843404.1	....
WP_147804655.1	....
WP_168007760.1	....
WP_146817052.1	....
WP_026691136.1	....
WP_171051829.1	....
WP_168009413.1	....
WP_124221886.1	....
WP_134339482.1	....
WP_091776380.1	....
WP_100334247.1	PGPGKPGNGKGNGRN
S08.133_sub._LD-1	....
WP_084380659.1	SEPTKPGNGKGNGRN
WP_134339480.1	....
WP_091776383.1	....
WP_146817050.1	....
WP_188208160.1	....
WP_146817048.1	....
WP_035661169.1	....
WP_027965007.1	....
WP_202078324.1	....
WP_129080804.1	....
WP_100374144.1	....
WP_110612024.1	....
WP_230895209.1	....
WP_075683870.1	....
WP_047973355.1	....
WP_138811387.1	....
WP_122897711.1	....
WP_101331250.1	....
WP_216831833.1	....
WP_226516443.1	....
WP_144089130.1	....
WP_096188791.1	....
WP_202080138.1	....
WP_167261846.1	....
WP_027963976.1	....
WP_091776386.1	....
WP_134338579.1	....
WP_101332746.1	....
WP_163537364.1	KKPKK..........
WP_099092793.1	K..............
WP_164853199.1	....
WP_090774843.1	....
WP_090775603.1	....
WP_059104808.1	....
WP_035392836.1	....
WP_078393865.1	....
EZH65969.1	....
KMK76635.1	....
WP_143850013.1	....
WP_203087429.1	....
WP_059105057.1	....
S08.098_sub._sendai	....
WP_003321226.1	....
WP_034632645.1	....
S08.157_sub._YaB	....
WP_060704798.1	....
WP_095239263.1	....
S08.010_M-pep.	....
S08.038_PB92	....
S08.003_Savinase	....
S08.028_ALTP	....
WP_143849870.1	....
WP_203088820.1	....
WP_017729072.1	....
WP_122896828.1	....
WP_047973137.1	....
S08.045_sub._ALP_1	....
WP_022628745.1	....
S08.046_sub._aprM	....
WP_053432556.1	....
WP_210595747.1	....
WP_129077943.1	....
WP_100374143.1	....
WP_216831504.1	....
WP_078596166.1	....
WP_035666680.1	....
WP_078597775.1	....
WP_100832725.1	....
WP_090774498.1	....
WP_096186536.1	....
WP_110520788.1	....
WP_122900894.1	....
WP_026691049.1	....
WP_199800957.1	....
S08.005_endopep._Q	....
CAO03040.1	....
WP_081105403.1	....
S08.034_sub._BPN’	....
WP_003155195.1	....
WP_003327717.1	....
WP_039073463.1	....
S08.002_mesentericopep.	....
S08.042_amylosacchariticus	....
S08.035_sub._J	....
S08.036_sub._E	....
S08.044_sub._NAT	....
WP_053604255.1	....
NPC92104.1	....

```
S08.001_sub._Carlsberg      ...............
AAS86761.1                  ...............
S08.037_sub._DY             ...............
NUJ19608.1                  ...............
WP_188377243.1              ...............
WP_173918387.1              ...............
KGX83542.1                  ...............
WP_051255158.1              ...............
WP_231417544.1              ...............
WP_096155439.1              ...............
AST90329.1                  ...............
WP_230500539.1              ...............
WP_078381234.1              ...............
WP_060666810.1              ...............
WP_152444042.1              ...............
WP_066412694.1              ...............
WP_230500606.1              ...............
WP_088017821.1              ...............
WP_224838688.1              ...............
WP_100334303.1              ...............
WP_094921089.1              ...............
WP_224844257.1              ...............
WP_070119644.1              ...............
WP_181472841.1              ...............
WP_136946078.1              ...............
WP_153236691.1              ...............
WP_077360649.1              ...............
WP_206945444.1              ...............
WP_029565418.1              ...............
TDL80277.1                  ...............
WP_224844255.1              ...............
WP_193538138.1              ...............
WP_078544469.1              ...............
WP_090849877.1              ...............
WP_022794977.1              ...............
WP_107586282.1              ...............
WP_106589713.1              ...............
WP_018922084.1              ...............
```

Fig. S18 MSA of mature subtilisin sequences of the S8 Bacillacea data mining sequences
