# Supplementary material for: Phylogenetic survey of the subtilase family and a data-mining-based search for new subtilisins from Bacillaceae
Source: Front Microbiol. 2022 Sep 26;13:1017978. doi: 10.3389/fmicb.2022.1017978 (PMC9549277; doi:10.3389/fmicb.2022.1017978)
Supplement: Supplementary file 2 [file Data_Sheet_2.docx]

# Supplementary


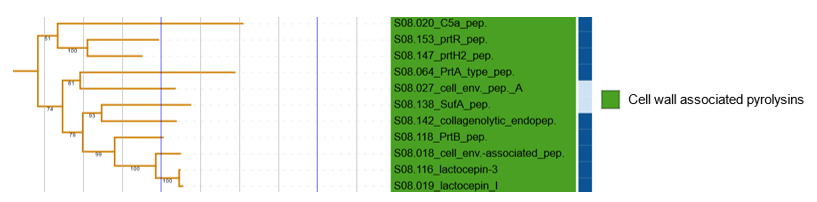


Fig. S1 Tree of cell wall associated subgroup (pyrolysin) of MEROPS holotype dataset


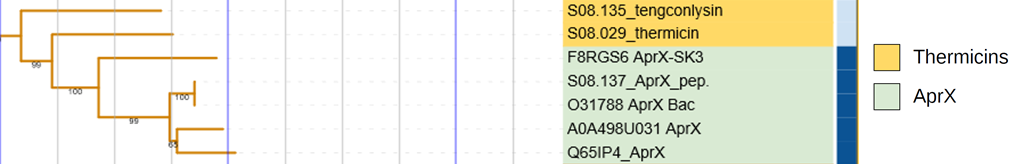


Fig. S2 Tree of thermicin and AprX subgroup (pyrolysin) of MEROPS holotype dataset


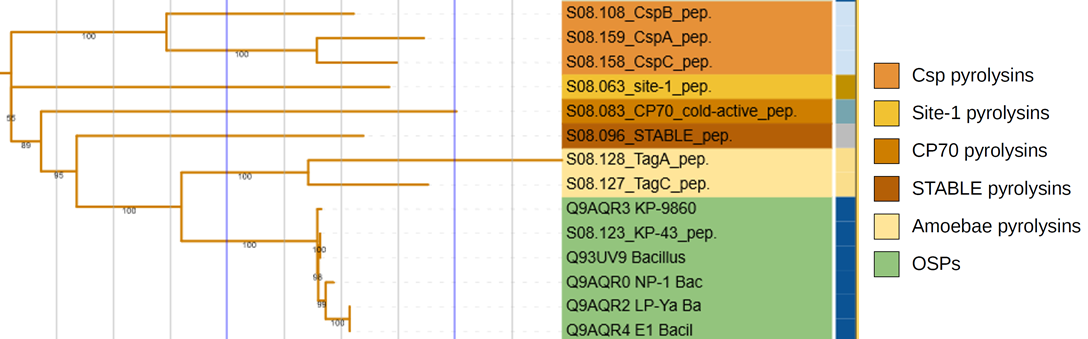


Fig. S3 Tree of Csp, Site-1, CP70, STABLE, Amoebae and OSP (pyrolysin) of MEROPS holotype dataset


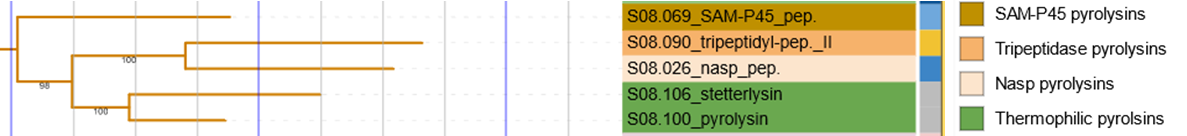


Fig. S4 Tree of SAM-P45, TPPII, Nasp and pyrolysin subgroup (pyrolysin) of MEROPS holotype dataset


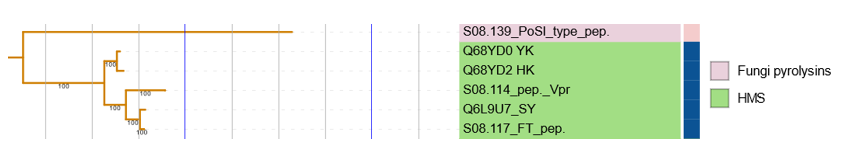


Fig. S5 Tree of fungal pyrolysins and HMS subgroup (pyrolysin) of MEROPS holotype dataset


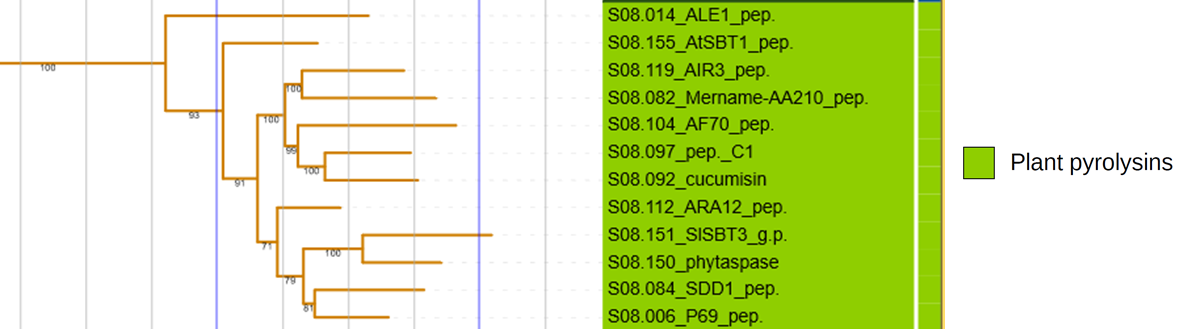


Fig. S6 Tree of plant subgroup (pyrolysin) of MEROPS holotype dataset


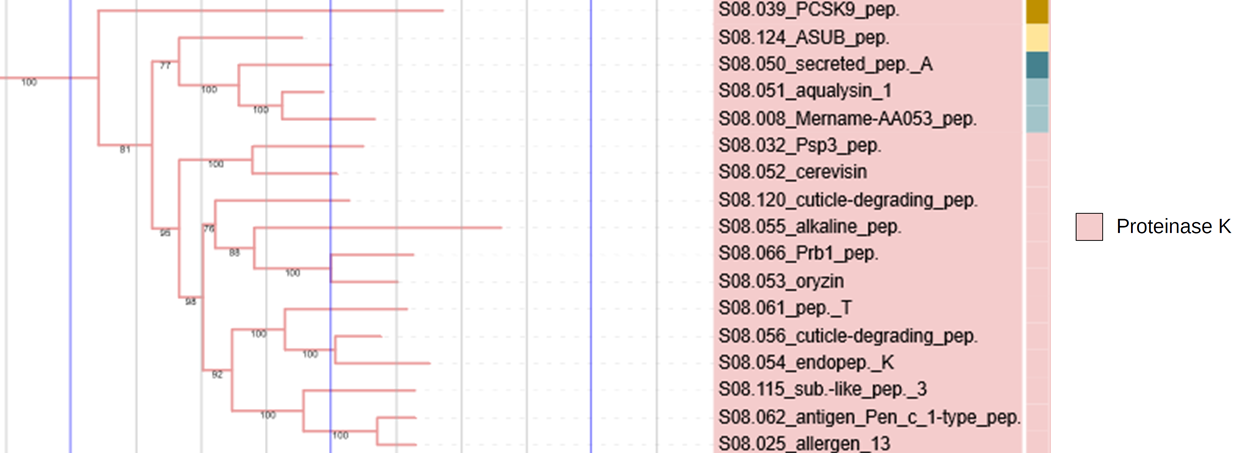


Fig. S7 Tree of proteinase K group of MEROPS holotype dataset


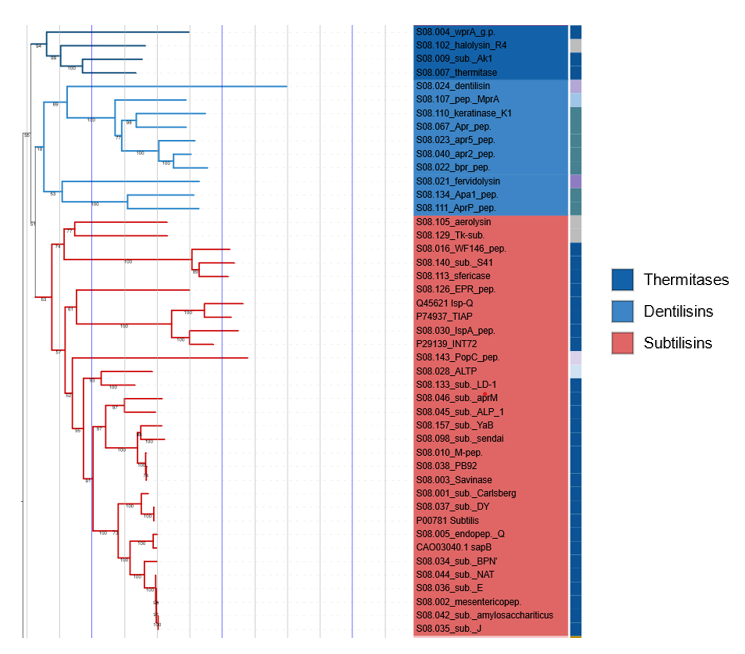


Fig. S8 Tree of subtilisin, dentilisin and thermitase group of MEROPS holotype dataset


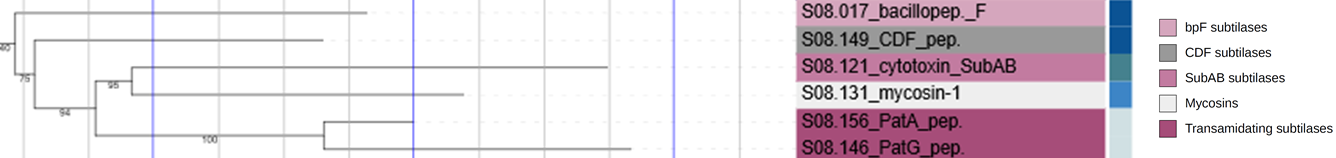


Fig. S9 Tree of bpF, CDF, SubAB, mycosins and transamidating groups of MEROPS holotype dataset


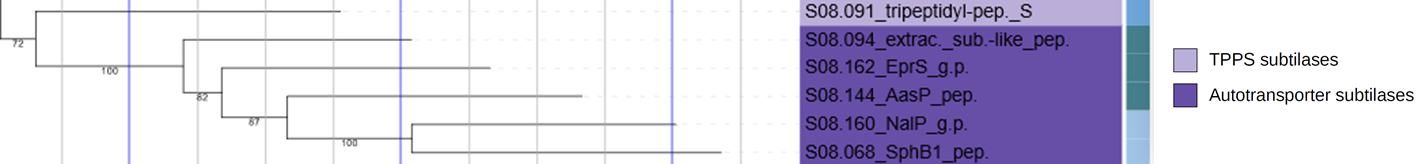


Fig. S10 Tree of TPPS and autotransporter group of MEROPS holotype dataset


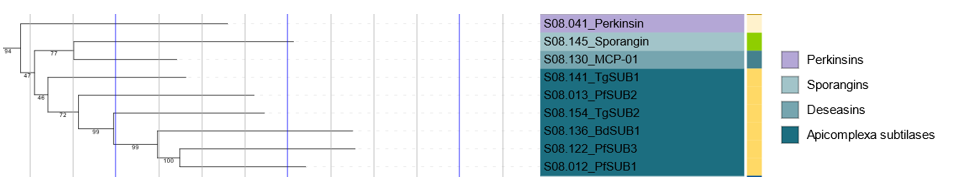


Fig. S11 Tree of perkinsin, sporangin, deseasin and apicomplexa group of MEROPS holotype dataset


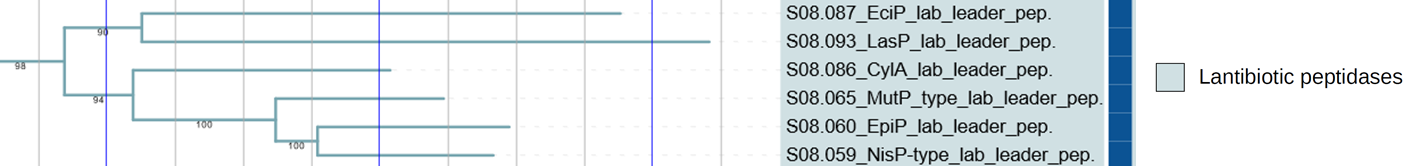


Fig. S12 Tree of lantibiotic peptidase group of MEROPS holotype dataset


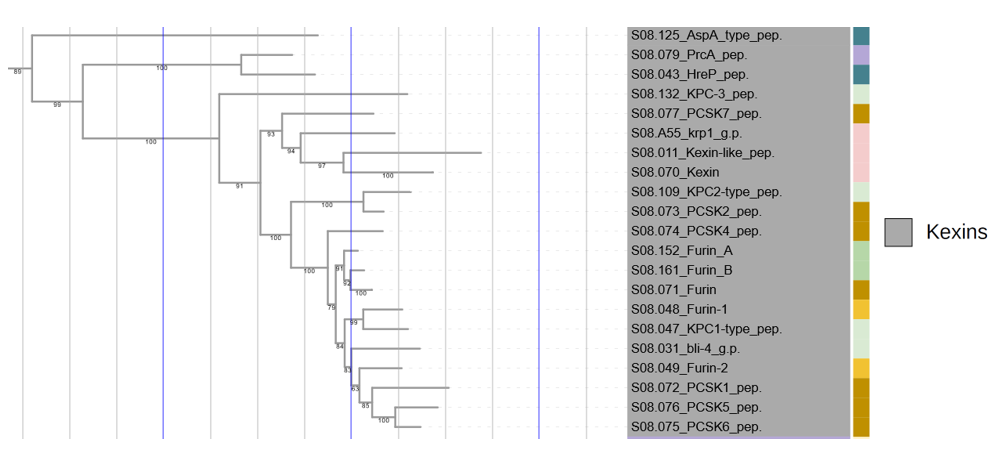


Fig. S13 Tree of kexin group of MEROPS holotype dataset


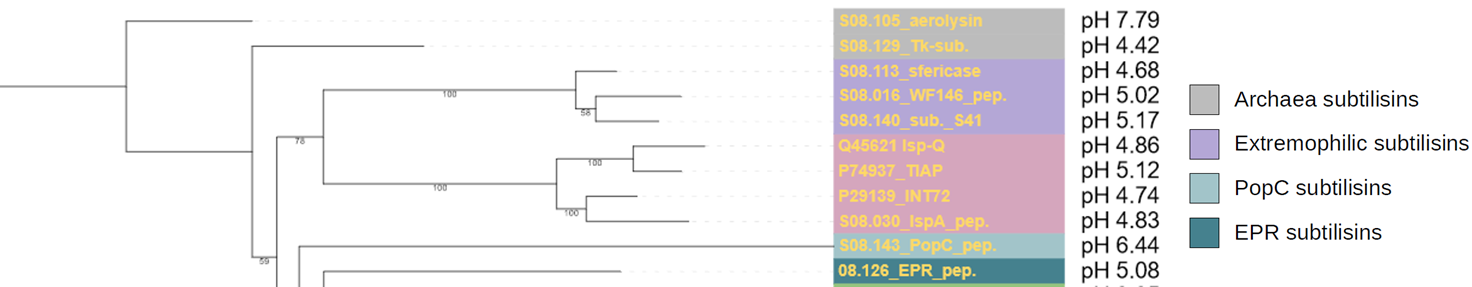


Fig. S14 Tree of several subgroups of subtilisin group of MEROPS holotype dataset


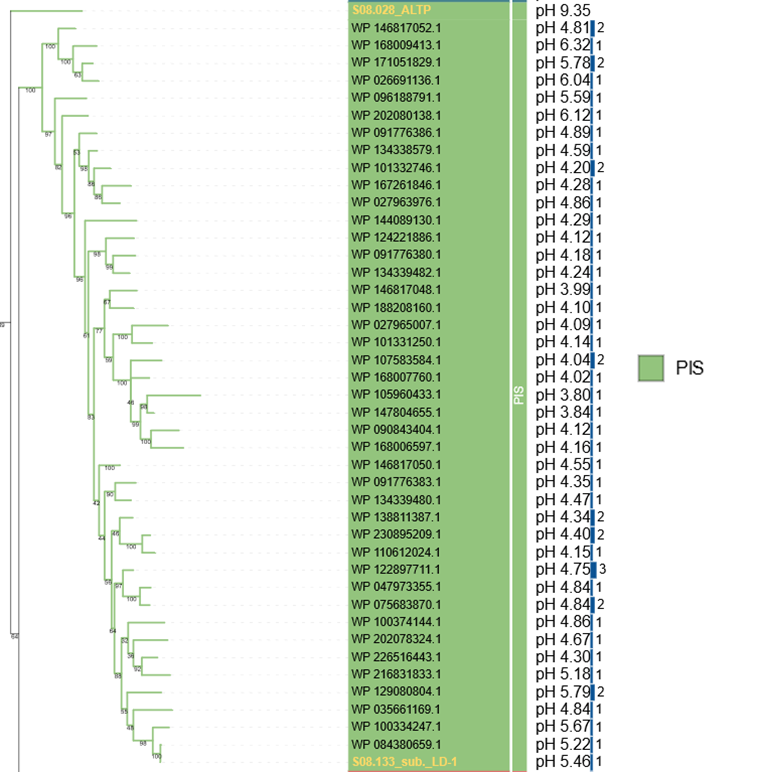


Fig. S15 Tree of PIS subgroup of subtilisin group


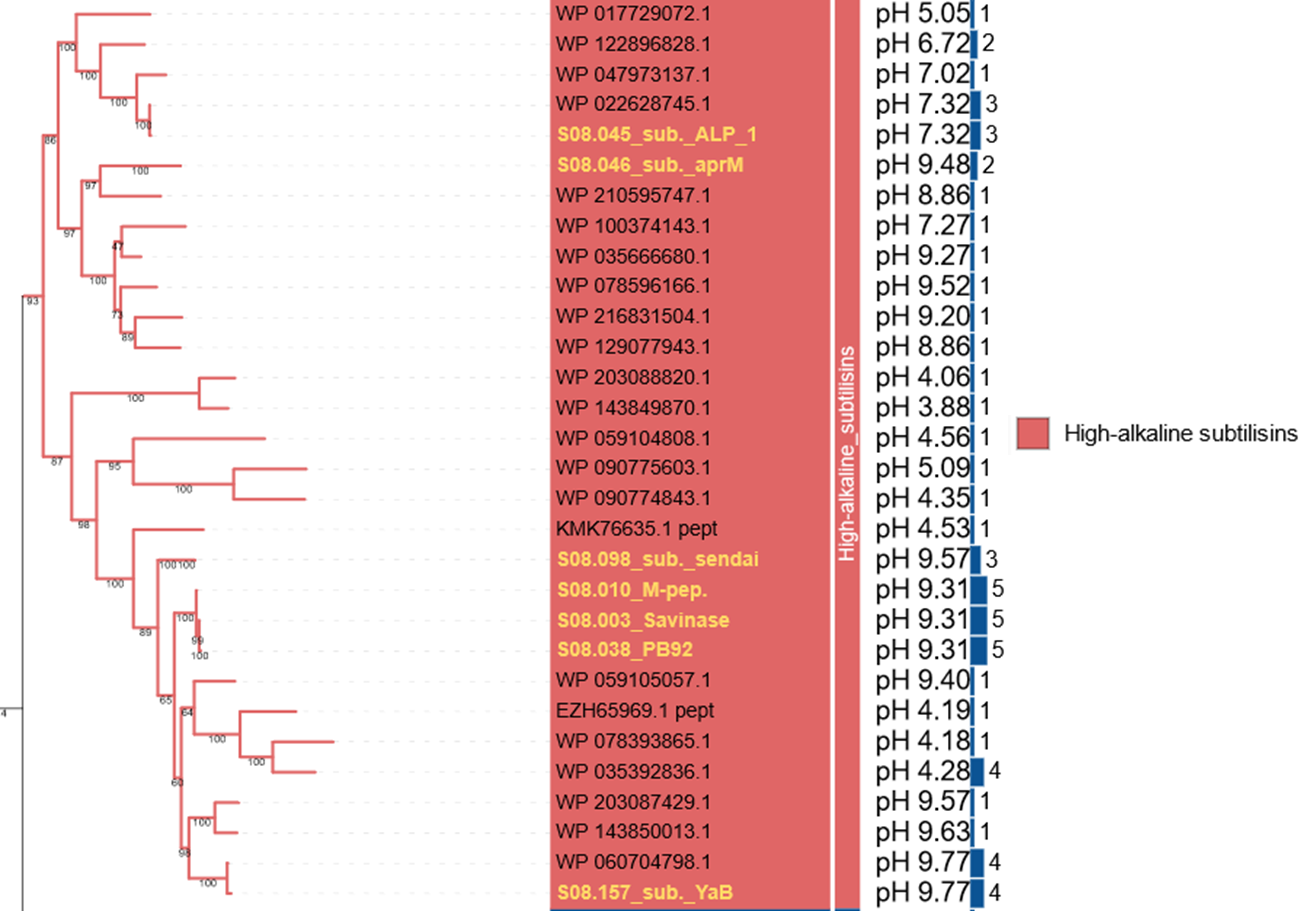


Fig. S16 Tree of high-alkaline subtilisins subgroup of subtilisin group


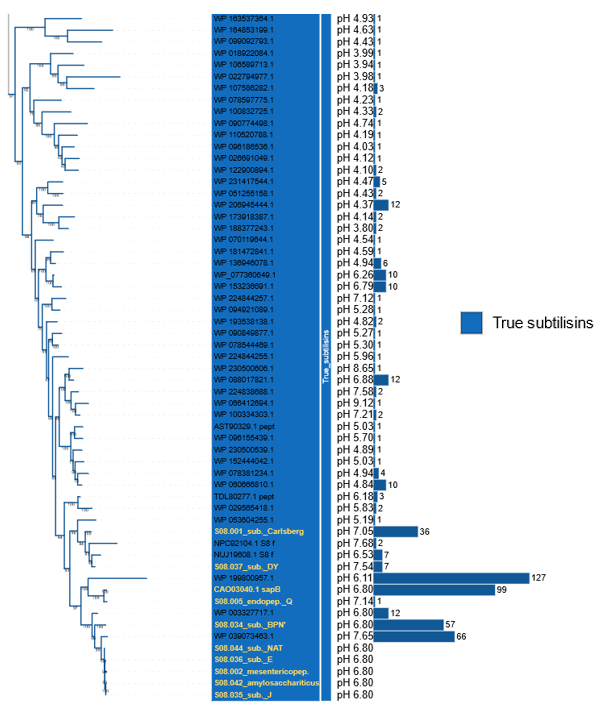


Fig. S17 Tree of true subtilisins subgroup of subtilisin group
