# Supplementary material for: Phylogenetic survey of the subtilase family and a data-mining-based search for new subtilisins from Bacillaceae
Source: Front Microbiol. 2022 Sep 26;13:1017978. doi: 10.3389/fmicb.2022.1017978 (PMC9549277; doi:10.3389/fmicb.2022.1017978)
Supplement: Supplementary file 3 [file Table_1.DOCX]

Table S1 Clustered data mining S8 *Bacillaceae* sequences

| Number # | *Bacterial origin* |
| --- | --- |
| S08.105_aerolysin | *Pyrobaculum aerophylum* |
| S08.129_Tk-sub. | *Thermococcus kodakarensis* |
| S08.113_sfericase | *Lysinibacillus sphaericus* |
| S08.016_WF146_pep. | *Bacillus* sp. WF146 |
| S08.140_sub._S41 | *Bacillus subtilis* TA41 |
| P29139_INT72 | *Paenibacillus polymyxa* |
| S08.030_IspA_pep. | *Bacillus subtilis* |
| Q45621 Isp-Q | *Bacillus* sp. NKS-21 |
| P74937_TIAP | *Thermoactinomyces sp.* |
| S08.143_PopC_pep. | *Myxococcus xanthus* |
| 08.126_EPR_pep. | *Bacillus subtilis* |
| S08.028_ALTP | *Alkaliphilus transvaalensis* |
| WP 146817052.1 | *Alkalibacillus haloalkaliphilus* |
| WP 168009413.1 | *Alkalicoccus luteus* |
| WP 171051829.1 | *Alteribacter natronophilus* |
| WP 026691136.1 | *Alteribacter aurantiacus* |
| WP 096188791.1 | *Bacillus* sp. FJAT 44876 |
| WP 202080138.1 | *Bacillus* sp. YIM B00319 |
| WP 091776386.1 | *Piscibacillus halophilus* |
| WP 134338579.1 | *Filobacillus milosensis* |
| WP 101332746.1 | *Halalkalibacillus sediminis* |
| WP 167261846.1 | *Alkalibacillus almallahensis* |
| WP 027963976.1 | *Halalkalibacillus halophilus* |
| WP 144089130.1 | *Allobacillus salarius* |
| WP 124221886.1 | *Aquisalibacillus elongatus* |
| WP 091776380.1 | *Piscibacillus halophilus* |
| WP 134339482.1 | *Filobacillus milosensis* |
| WP 146817048.1 | *Alkalibacillus haloalkaliphilus* |
| WP 188208160.1 | *Alkalibacillus aidingensis* |
| WP 027965007.1 | *Halalkalibacillus halophilus* |
| WP 101331250.1 | *Halalkalibacillus sediminis* |
| WP 107583584.1 | *Alkalicoccus saliphilus* |
| WP 168007760.1 | *Alkalicoccus luteus* |
| WP 090843404.1 | *Alkalicoccus daliensis* |
| WP 168006597.1 | *Alkalicoccus luteus* |
| WP 105960433.1 | *Alkalicoccus urumqiensis* |
| WP 147804655.1 | *Alkalicoccus halolimnae* |
| WP 146817050.1 | *Alkalihalobacillus haloalkaliphilus* |
| WP 091776383.1 | *Piscibacillus halophilus* |
| WP 134339480.1 | *Filobacillus milosensis* |
| WP 138811387.1 | *Alteribacter natronophilus* |
| WP 230895209.1 | *Salipaludibacillus* sp. CUR1 |
| WP 110612024.1 | *Salipaludibacillus keqinensis* |
| WP 122897711.1 | *Bacillus* sp. KQ 3 |
| WP 047973355.1 | *Bacillus* sp. LL01 |
| WP 075683870.1 | *Alkalihalobacillus pseudofirmus* |
| WP 100374144.1 | *Bacillus* sp. FJAT 45037 |
| WP 202078324.1 | *Bacillus* sp. YIM B00319 |
| WP 226516443.1 | *Bacillus shivajii* |
| WP 216831833.1 | *Alkalihalobacterium elongatum* |
| WP 129080804.1 | *Anaerobacillus alkaliphilus* |
| WP 035661169.1 | *Halalkalibacter akibai* |
| WP 100334247.1 | *Bacillus alkalisoli* |
| WP 084380659.1 | *Sutcliffiella cohnii* |
| S08.133_sub._LD-1 | *Bacillus* sp. KSM-LD1 |
| WP 017729072.1 | *Halalkalibacterium ligniniphilus* |
| WP 122896828.1 | *Bacillus* sp. KQ 3 |
| WP 047973137.1 | *Bacillus* sp. LL01 |
| WP 022628745.1 | *Alkalihalophilus marmarensis* |
| S08.045_sub._ALP_1 | *Bacillus* sp. nks-21 |
| WP 210595747.1 | *Bacillus* sp. YZJH907 2 |
| S08.046_sub._aprM | *Halalkalibacterium halodurans* |
| WP 100374143.1 | *Bacillus* sp. FJAT 45037 |
| WP 035666680.1 | *Alkalihalobacillus akibai* |
| WP 078596166.1 | *Evansella clarkii* |
| WP 216831504.1 | *Alkalihalobacterium elongatum* |
| WP 129077943.1 | *Anaerobacillus alkaliphilus* |
| WP 203088820.1 | *Shouchella gibsonii* |
| WP 143849870.1 | *Bacillus* sp. P16 2019 |
| WP 059104808.1 | *Shouchella shacheensis* |
| WP 090775603.1 | *Alkalihalobacillus lonarensis* |
| WP 090774843.1 | *Alkalihalobacillus lonarensis* |
| KMK76635.1 pept | *Alkalihalobacillus pseudalcaliphilus* |
| WP 034632645.1 | *Alkalihalobacillus okhensis* |
| S08.098_sub._sendai | *Bacillus* sp. G-825-6 |
| S08.010_M-pep. | *Shouchella clausii* |
| S08.003_Savinase | *Lederbergia lentus* |
| S08.038_PB92 | *Alkalihalobacillus alcalophilus* |
| WP 060704798.1 | *Shouchella miscanthi* |
| S08.157_sub._YaB | *Bacillus* sp. YAB |
| WP 203087429.1 | *Shouchella gibsonii* |
| WP 143850013.1 | *Bacillus* sp. P16 2019 |
| WP 059105057.1 | *Shouchella shacheensis* |
| EZH65969.1 pept | *Bacillaceae bacterium JMAK1* |
| WP 078393865.1 | *Shouchella patagoniensis* |
| WP 035392836.1 | *Bacillus* sp. JCM19047 |
| WP 163537364.1 | *Gracilibacillus sp. YIM 98692* |
| WP 164853199.1 | *Peribacillus asahii* |
| WP 099092793.1 | *Bacillus weihaiensis* |
| WP 018922084.1 | *Salsuginibacillus kocurii* |
| WP 106589713.1 | *Salsuginibacillus halophilus* |
| WP 022794977.1 | *Marinococcus halotolerans* |
| WP 107586282.1 | *Alkalicoccus saliphilus* |
| WP 078597775.1 | *Evansella clarkii* |
| WP 100832725.1 | *Bacillus* sp. FJAT 45348 |
| WP 090774498.1 | *Alkalihalobacillus lonarensis* |
| WP 110520788.1 | *Bacillus lacisalsi* |
| WP 096186536.1 | *Bacillus* sp. FJAT 44876 |
| WP 026691049.1 | *Alteribacter aurantiacus* |
| WP 122900894.1 | *Bacillus* sp. KQ 3 |
| WP 231417544.1 | *Pontibacillus* sp. HN14 |
| WP 051255158.1 | *Pontibacillus marinus* |
| WP 206945444.1 | *Halobacillus* sp. GSS1 |
| WP 173918387.1 | *Halobacillus* sp. Marseille-Q1614 |
| WP 188377243.1 | *Halobacillus andaensis* |
| WP 070119644.1 | *Bacillus marinisedimentorum* |
| WP_077360649.1 | *Fictibacillus arsenicus* |
| WP 153236691.1 | *Fictibacillus phosphorivorans* |
| WP 181472841.1 | *Halobacillus locisalis* |
| WP 136946078.1 | *Pseudalkalibacillus hwajinpoensis* |
| WP 224844257.1 | *Bacillus timonensis* |
| WP 094921089.1 | *Lottiidibacillus patelloidae* |
| WP 193538138.1 | *Cytobacillus luteolus* |
| WP 090849877.1 | *Litchfieldia salsus* |
| WP 078544469.1 | *Litchfieldia alkalitelluris* |
| WP 224844255.1 | *Bacillus timonensis* |
| WP 230500606.1 | *Bacillus* sp. JJ 125 |
| WP 088017821.1 | *Sutcliffiella horikoshii* |
| WP 224838688.1 | *Bacillus tianshenii* |
| WP 066412694.1 | *Sutcliffiella cohnii* |
| WP 100334303.1 | *Bacillus alkalisoli* |
| AST90329.1 pept | *Sutcliffiella cohnii* |
| WP 096155439.1 | *Bacillus alkalisoli* |
| WP 230500539.1 | *Bacillus* sp. JJ 125 |
| WP 152444042.1 | *Bacillus* sp. THAF10 |
| WP 078381234.1 | *Sutcliffiella halmapala* |
| WP 060666810.1 | *Bacillus* sp. CHD6a |
| TDL80277.1 pept | *Brevibacterium frigoritolerans* |
| WP 029565418.1 | *Metabacillus indicus* |
| WP 053604255.1 | *Bacillus gobiensis* |
| AAS86761.1_kera | *Bacillus licheniformis* |
| S08.001_sub._Carlsberg | *Bacillus licheniformis* |
| NPC92104.1 S8 f | *Bacillus* sp. WMMC1349 |
| NUJ19608.1 S8 f | *Bacillus glycinifermentans* |
| S08.037_sub._DY | *Bacillus subtilis* |
| WP 199800957.1 | *Bacillus pumilus* |
| S08.005_endopep._Q | *Bacillus pumilus* |
| CAO03040.1 sapB | *Bacillus pumilus* |
| WP 003327717.1 | *Bacillus atrophaeus* |
| S08.034_sub._BPN' | *Bacillus amyloliquefaciens* |
| WP 039073463.1 | *Bacillus vallismortis* |
| S08.044_sub._NAT | *Bacillus subtilis* |
| S08.036_sub._E | *Bacillus subtilis* |
| S08.002_mesentericopep. | *Bacillus pumilus* |
| S08.042_amylosacchariticus | *Bacillus subtilis* |
| S08.035_sub._J | *Geobacillus stearothermophilus* |
